# The Inclusion of Dietary and Medicinal Mushrooms into Translational Oncology: Pros and Cons at the Molecular Level

**DOI:** 10.3390/ijms27031312

**Published:** 2026-01-28

**Authors:** Yulia Kirdeeva, Elizaveta Fefilova, Natalia Karpova, Sergey Parfenyev, Alexandra Daks, Alexander Nazarov, Oleg Semenov, Nguyen Thi Van Anh, Vu Thanh Loc, Nguyen Manh Cuong, Oleg Shuvalov

**Affiliations:** 1 Institute of Cytology, Russian Academy of Sciences, 194064 St. Petersburg, Russia; yulia.kirdeeva@yandex.ru (Y.K.); e.fefilova@list.ru (E.F.); natalyndk@gmail.com (N.K.); gen21eration@gmail.com (S.P.); alexandra.daks@gmail.com (A.D.); anazarov2021@gmail.com (A.N.); semyonov.somspb@yandex.ru (O.S.); 2 Department of Life Sciences, University of Science and Technology of Hanoi, Vietnam Academy of Science and Technology (VAST), 18 Hoang Quoc Viet, Nghia Do, Hanoi 11353, Vietnam; vananh.pharm@gmail.com; 3 Center for Development of Natural Products and Technology—Equipments, Institute of Chemistry, Vietnam Academy of Science and Technology (VAST), 18 Hoang Quoc Viet, Nghia Do, Hanoi 11353, Vietnam; vuloc1110@gmail.com

**Keywords:** mushroom, polysaccharides, β-glucans, signaling pathways, immunomodulation, low-molecular-weight compounds

## Abstract

Mushrooms are valued for their nutritional qualities and have been used in traditional medicine since the Neolithic era. They exhibit various bioactivities, including antioxidant, hypocholesterolemic, immunomodulatory, and anticancer effects. The anticancer effects arise via direct action on tumor cells and indirect modulation of the immune system; the latter is the predominant mechanism. Numerous studies indicate that various mushroom species are potent immunostimulants because their cell wall polysaccharides and proteoglycans are recognized by intestinal immune cells. This enhances antitumor immunity through multiple molecular pathways. However, their direct effects on cancer cells are of questionable physiological relevance due to bioavailability constraints. Nevertheless, we hypothesize that the accumulation of non-absorbed polysaccharides in the gastrointestinal tract positions mushrooms as dual-action agents with the potential to treat colorectal cancer by providing indirect immunomodulation and direct local tumor suppression. Conversely, the direct anticancer effects of mushrooms are generally attributed to bioactive secondary metabolites that influence essential cellular processes, including signaling pathways, cell cycle regulation, apoptosis, autophagy, cellular migration, invasion, and cancer stem cell characteristics. Beyond these anticancer effects, clinical evidence suggests that certain mushroom-derived substances can improve survival outcomes for cancer patients and provide supportive care benefits in oncology, thereby improving quality of life. Specifically, mushrooms may mitigate the side effects of chemotherapy and radiotherapy, bolster immune function often suppressed by cancer treatments, and enhance overall well-being. In this review, we discuss the therapeutic benefits of dietary and medicinal mushrooms in cancer care, as well as unresolved challenges and future research directions.

## 1. Introduction

Natural compounds from plants and mushrooms are a source of continuous development for anticancer therapeutics [1,2,3,4,5]. According to the literature, approximately 40,000 species of mushrooms exist worldwide, 2189 of which are edible [6]. Mushrooms are low in calories and fat and are rich in insoluble fiber. They are also a non-animal source of high-quality protein, vitamins (particularly B and D), minerals, and other beneficial components [7]. Three species of mushrooms that are now cultivated worldwide are white button mushrooms (*Agaricus bisporus*), oyster mushrooms (*Pleurotus ostreatus*), and paddy straw mushrooms (*Volvariella volvacea*). The Romans considered these mushrooms to be the “food of the gods,” and the Chinese considered them to be the “elixir of life” [8].

Beyond their dietary properties, mushrooms contain diverse and potent bioactive compounds, making them useful for medical purposes [9]. Although the medicinal properties of mushrooms have been recognized for centuries and several species are prominent in traditional Chinese medicine (TCM), Ayurveda, and Kampo, we believe the benefits of consuming mushrooms are significantly underestimated. A deep, systematic scientific study of their medical properties is required.

Indeed, there are several hundred publications describing the various valuable biological properties of mushrooms, including their antioxidant, anti-inflammatory, anticancer, and cholesterol-lowering properties (reviewed in [9,10,11,12]). Based on this extensive literature, we can confidently conclude that the primary medical property of mushrooms is their immunoregulatory function, a trait common to different, unrelated species. The strong biological activity of mushrooms appears to be closely linked to the structural components of their cell walls, such as polysaccharides (e.g., β-glucans) and proteoglycans. Recognized as pathogen-associated molecular patterns (PAMPs) in the intestine, β-glucans and proteoglycans boost innate immunity and enhance the adaptive immune response to different antigens. This results in enhanced immunity against various stimuli, including anticancer immunity [13].

In addition to polysaccharides, which significantly boost anticancer immunity, mushrooms contain secondary metabolites with various biological activities that contribute to their antineoplastic properties. The most abundant of these compounds are terpenes, followed by phenolic constituents and other minor types. While some affect the immune system, they typically exhibit antineoplastic properties by directly impacting hallmarks of cancer cells. They typically downregulate common oncogenes and inhibit signaling pathways, inducing cell cycle arrest and apoptosis while impeding migration and invasion capabilities.

Finally, some mushroom-derived peptides known as fungal immunomodulating proteins (FIPs) have strong antineoplastic properties. These peptides boost the immune system while inhibiting signaling processes in malignant cells.

In this review, we present two central theses for critically appraising the field. First, the systemic anticancer efficacy of mushroom-derived macromolecules (polysaccharides/proteoglycans) is predominantly mediated by indirect immunomodulation. Their direct effects on cancer cells are of questionable physiological relevance due to bioavailability constraints. However, this direct action may hold specific promise for colorectal cancer. Second, in contrast, the direct antineoplastic potential of mushrooms resides chiefly in their low-molecular-weight metabolites, which target core oncogenic pathways. However, their translational development requires rigorous prioritization based on mechanistic clarity and preclinical robustness.

Finally, we will discuss the safety, limitations, and clinical experience associated with the use of medical mushrooms in oncology.

## 2. Health Benefits and Antineoplastic Activity of Edible and Medical Mushrooms at a Glance

Of the more than 14,000 species of mushrooms, 270 have been identified as providing health benefits to humans [14]. Edible mushrooms have long been a popular food source in various regions, particularly in Asia. In recent years, their consumption has increased worldwide.

**Figure 1:**
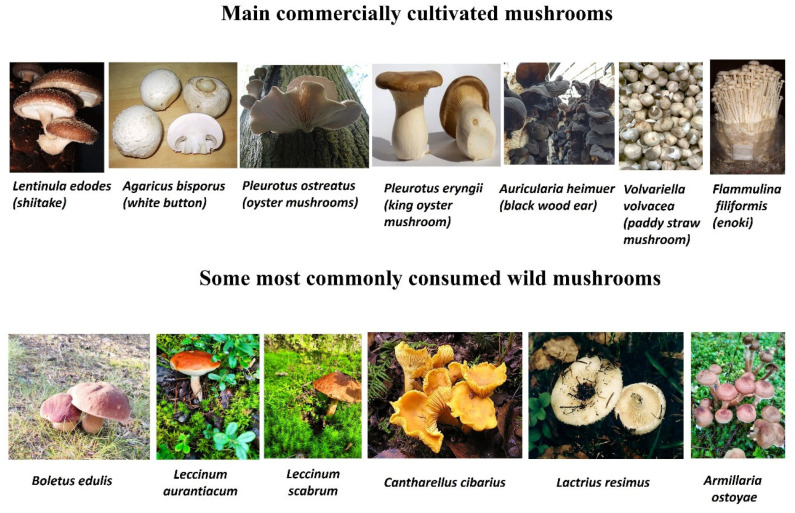
The examples of the main commercially cultivated and most commonly consumed wild mushrooms. The photos presented were taken by the authors, as well as photos from the open source (Wikipedia).

First, consumption has increased due to the commercial cultivation of mushrooms. First, the consumption of mushrooms has increased due to their commercial cultivation. The most commercially important species are *Agaricus bisporus* (white button mushrooms), *Lentinula edodes* (shiitake mushrooms), *Pleurotus ostreatus* (oyster mushrooms), *Pleurotus eryngii* (king trumpet mushroom or king oyster mushroom), *Auricularia heimuer* (black wood ear), *Volvariella volvacea* (paddy straw mushroom), and *Flammulina filiformis* (enokitake mushrooms) (Figure 1).

Secondly, wild mushrooms are a popular food in several countries, especially in Europe. The most commonly consumed wild mushrooms belong to the following genera: *Boletus*, *Leccinum*, *Cantharellus*, *Lactarius*, and *Armillaria* (Figure 1).

Various studies have demonstrated the positive impact of consuming mushrooms on human health. In addition to their nutritional value, mushrooms have a long history of use in traditional medicine. They possess immunomodulatory, antineoplastic, and cholesterol-lowering properties. Immunomodulatory activity is central and includes immunostimulatory and anti-inflammatory properties [9]. The well-documented immunostimulatory activity is characteristic of various mushroom species. These same species are also known for their anti-inflammatory properties against various inflammatory disorders, including allergies, ulcerative colitis, and psoriasis [13].

Immunomodulatory activity is closely associated with the potent antineoplastic properties of mushrooms [15,16,17]. Several meta-analysis studies have shown that higher mushroom consumption is associated with a decreased cancer risk [18,19,20]. For instance, a meta-analysis of dose–response studies from 1966 to 2020 revealed that individuals who consumed about 18 g of mushrooms daily were 45% less likely to develop cancer than non-consumers [18].

Many (if not all) mushroom species possess immunomodulatory and antineoplastic properties due to the presence of polysaccharides and proteoglycans in their cell walls [13]. This may explain the observed association between higher general mushroom consumption and lower cancer incidence.

**Figure 2:**
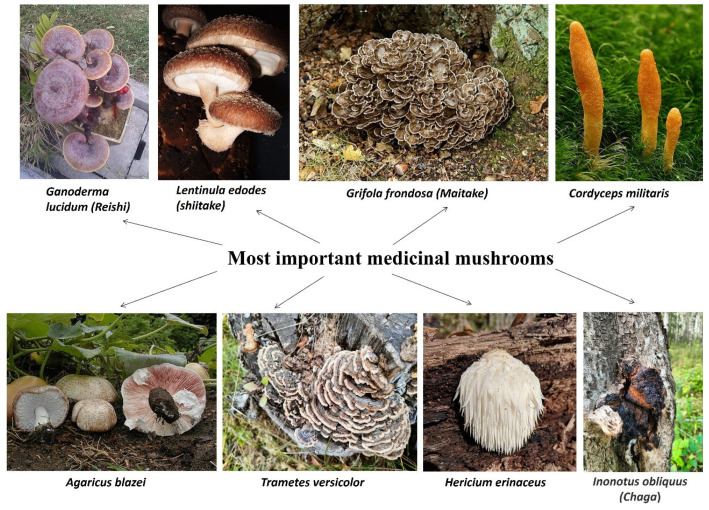
The major medicinal mushrooms with well-known anticancer properties. The photos presented were taken by the authors, as well as photos from the open source (Wikipedia).

However, antitumor properties have been documented in approximately 100 species of mushrooms, including edible and medicinal varieties [21]. Below, we list the most important medicinal mushrooms and direct interested readers to excellent reviews on the topic: *Ganoderma lucidum* (Reishi) [22], *Lentinula edodes* (Shiitake) [23], *Grifola frondosa* (Maitake) [24], *Trametes versicolor* (Turkey tail) [25], *Trametes robiniophila*, or *Vanderbylia robiniophila* (Huaier) [26], *Hericium erinaceus* (Lion’s Mane) [27], *Inonotus obliquus* (Chaga) [28], and *Agaricus blazei* (Sun Mushroom) [29] (Figure 2).

First, the antineoplastic properties of mushrooms appear to be conferred by polysaccharides and proteoglycans, followed by low-molecular-weight compounds, including terpenes and, to a lesser extent, phenolic compounds. Indeed, mushroom-derived glucans and proteoglycans are well-known immune boosters that strengthen antineoplastic immunity. Numerous studies have shown that these compounds affect both the innate and adaptive immune systems, resulting in stimulation and activation of dendritic cells (DCs), increased natural killer (NK) cell numbers and activity, B lymphocyte proliferation and differentiation, T lymphocyte activation, and elevated IFN-γ, TNF-α, and IL-12 levels [13]. They also restore the balance of Th1/Th2 lymphocytes (Tregs) and the granulocyte-lymphocyte ratio, as well as regulate the M1/M2 macrophage polarization ratio [1]. These effects fine-tune the immune system’s response to tumor cells.

Terpenes, polyphenols, and other low-molecular-weight compounds primarily affect cancer cells directly. These compounds generally affect multiple signaling pathways by inhibiting the proliferation, migration, and invasion of tumor cells, arresting the cell cycle, and inducing apoptosis.

The next chapters discuss the antineoplastic properties of mushroom-derived polysaccharides and low-molecular-weight compounds in detail.

## 3. Immunomodulation by Mushroom Polysaccharides: Harnessing the Anti-Tumor Immune Response

**Figure 3:**
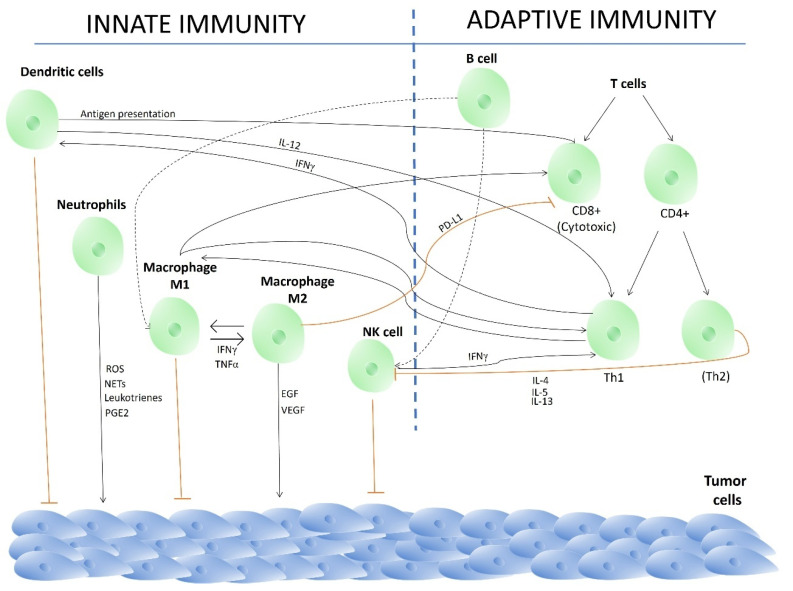
An overview of the interplay between innate and adaptive immunity in the defense against cancer. Dendritic cells (DCs) present antigens to CD8^+^ T cells. They are also crucial for initiating Th1 immune responses because they produce interleukin-12 (IL-12), which drives the differentiation of naïve T cells into Th1 cells. Th1 cells then activate DCs by producing IFN-γ, which enhances their ability to kill tumor cells directly. M1 macrophages promote Th1 cell development and function. Th1 cells can further polarize macrophages toward the M1 phenotype, creating a positive feedback loop. Additionally, M1 macrophages can induce and activate CD8^+^ T cells. In turn, M2 macrophages secrete vascular endothelial growth factor (VEGF) and epidermal growth factor (EGF). VEGF stimulates angiogenesis, and EGF activates the epidermal growth factor receptor (EGFR) signaling pathway. These factors can encourage proliferation and metastasis. Furthermore, M2 macrophages can promote PD-L1 expression on cancer cells, thereby reducing the ability of CD8^+^ T cells to kill tumor cells. Neutrophils produce reactive oxygen species (ROS), which increase the mutation load in malignant cells. They also secrete other pro-tumorigenic components, such as neutrophilic extracellular traps (NETs), leukotrienes, and prostaglandin E2 (PGE2). Natural killer (NK) cells destroy tumor cells and produce interferon gamma (IFNγ), which influences the differentiation of Th1 cells. Th2 cells secrete cytokines, including IL-4, IL-5, and IL-13, that promote the development of M2 macrophages. Additionally, IL-4, produced by Th2 cells, can indirectly enhance the metastatic potential of cancer cells. Tumor-infiltrating B cells produce tumor-specific antibodies that destroy tumors by attracting NK cells, macrophages, and neutrophils. The arrows indicate activation of processes, and the bars indicate suppression. Further explanations are provided in the text.

The immune system acts as a vital defense against cancer. It constantly patrols for abnormal cells and recognizes cancerous ones via tumor-specific (TSA) or tumor-associated antigens (TAAs) [30]. Then, it deploys white blood cells, such as T cells and natural killer (NK) cells, to destroy the cancerous cells. This process is called immunosurveillance [31]. While this defense is typically effective at eliminating early-stage cancers, tumors can evade it by “hiding” or releasing signals that suppress immune activity. Thus, the immune system plays a dual role in cancer. It can eliminate malignant cells but, when dysregulated, it can also promote tumor progression [32]. The balance between immune surveillance and tumor-promoting inflammation is orchestrated by a complex network of innate and adaptive immune cells within the tumor microenvironment (Figure 3).

Importantly, the evidence clearly demonstrates that the primary anticancer mechanism of many medicinal mushrooms is the strategic modulation of the immune network, rather than direct cytotoxicity. This chapter first outlines the key immune cells involved in anti-tumor immunity and immunosuppression. Then, it details how mushroom-derived polysaccharides and proteoglycans—most notably β-glucans—interact with this system. By engaging pattern recognition receptors on immune cells in the gut-associated lymphoid tissue (GALT), these compounds can reprogram the tumor microenvironment (TME), enhance immune surveillance, and counteract tumor-induced immunosuppression. Thus, they leverage the host’s own defenses to combat cancer.

### 3.1. The Immune System in Cancer: A Dual-Role Framework

The innate immune system is a vital first line of defense against cancer. This system includes natural killer (NK) cells, macrophages, neutrophils, and dendritic cells (DCs). However, tumors can develop escape mechanisms by co-opting components of the tumor microenvironment (TME), which can impair anti-tumor responses [33] (Figure 3).

NK cells rapidly eliminate tumor cells, particularly those that have lost HLA-I expression [34], and their abundance correlates with a favorable prognosis [35,36]. However, the tumor microenvironment (TME) can suppress their activity [37]. Macrophages exhibit plasticity. Classically activated M1 macrophages promote anti-tumor immunity, while alternatively activated M2 macrophages (often tumor-associated macrophages, TAMs) support angiogenesis, immunosuppression via PD-L1, and tumor progression [38,39,40,41]. Neutrophils are increasingly recognized for their pro-tumorigenic roles in promoting inflammation, immunosuppression, and angiogenesis. An elevated neutrophil-to-lymphocyte ratio is a poor prognostic indicator [42,43]. Dendritic cells (DCs) are essential for initiating adaptive anti-tumor immunity by presenting tumor antigens to T cells [44,45,46] (Figure 3). Their infiltration level can be prognostically significant [47].

The adaptive immune system provides specific antitumor immunity primarily through CD8+ cytotoxic T lymphocytes (CTLs). These cells recognize tumor antigens, including neoantigens and viral oncoproteins, which are presented by HLA class I molecules [48,49,50] (Figure 3). CD4^+^ T helper cells orchestrate this response. Th1 cells promote anti-tumor immunity by activating macrophages and CTLs via IFN-γ. In contrast, Th2 cells promote immunosuppression by inducing M2 macrophage polarization [51,52]. B cells can produce tumor-specific antibodies that mediate antibody-dependent cellular cytotoxicity (ADCC) [53].

In parallel, tumors evade this adaptive response through multiple complementary mechanisms. These include loss of target antigen expression [54], downregulation of HLA-I presentation via genetic or epigenetic alterations [55,56], secretion of anti-inflammatory cytokines [57], and upregulation of immune checkpoint ligands, such as PD-L1 [58]. These strategies disrupt the Th1/Th2 equilibrium, shifting the tumor microenvironment from an immunogenic (Th1) state to an immunosuppressive (Th2) state and facilitating tumor progression [59]. Therefore, a primary therapeutic objective is to restore and amplify the Th1/CTL axis while blocking these critical immune escape pathways.

In addition, cancer immune escape is conferred by MDSCs (myeloid-derived suppressor cells), which are immature immune cells that accumulate in cancer patients. MDSCs promote tumor growth by suppressing anti-tumor immunity and fostering metastasis, angiogenesis, and therapy resistance. MDSCs inhibit T cells, NK cells, and DCs, while promoting tumor cell stemness and epithelial–mesenchymal transition (EMT) [32].

### 3.2. Mushroom Polysaccharides as Immune System Modulators

#### 3.2.1. Key Bioactive Molecules: β-Glucans and Proteoglycans

Various mushroom-derived constituents derived from mushrooms have been shown to contribute to anti-cancer activity. However, cell wall-derived macromolecules, such as polysaccharides and proteoglycans, are primarily responsible for this activity. Among these polysaccharides, β-glucans, which are glucose polymers characterized by (1 → 3)-β and (1 → 6)-β linkages, are the most therapeutically significant. These polysaccharides have been shown to exhibit significant biological activity in mammals and humans [60,61,62].

Mushroom-derived α-glucans are less popular than β-glucans in cancer research, but they also possess antineoplastic properties. For example, active hexose correlated compound (AHCC), a rich source of alpha-glucans derived from shiitake mushrooms, is a prominent subject of cancer research and a widely used alternative medicine in Japan. It is being investigated for its potential as an anti-cancer agent [63]. The highly branched α-glucan YM-2A, found in maitake mushrooms, activates immune cells in the Peyer’s patches of the gut, including dendritic cells and macrophages, thereby exerting its antitumor effects. This local activation induces a systemic antitumor immune response, characterized by an increase in CD4^+^ and CD8^+^ T cells that produce interferon-gamma (INF-γ). This leads to inhibited tumor growth and improved survival in mouse models of colon carcinoma and melanoma [64]. Similarly, the marine-derived branched α-D-glucan JNY2PW enhances tumor cell sensitivity to immune attack by downregulating the chemokine CXCL5 in cancer cells via the Akt/mTOR and ERK/GSK3β/β-catenin pathways. It also inhibits tumor proliferation and epithelial–mesenchymal transition (EMT) *in vivo* without apparent toxicity [65]. Taken together, these findings suggest that alpha-glucans are promising oral immunomodulatory adjuvants for cancer immunotherapy.

A proteoglycan is a more complex molecule in which a core protein is covalently attached to one or more carbohydrate chains. Proteoglycans are not as prevalent or defined in the same way in mushrooms and other fungi as they are in animals. A more accurate and functionally equivalent term for these molecules is “fungal proteoglycans.” Unlike animal-derived proteoglycans, the heavily glycosylated proteins in fungi are not decorated with glycosaminoglycans. Instead, they are modified with various types of highly branched polysaccharide complexes, primarily β-glucans and mannans, which are covalently linked to the protein core [66].

The two most famous and clinically important mushroom-derived proteoglycans are PSK (polysaccharide K, or Krestin) and PSP (polysaccharide peptide), both of which are derived from *Trametes versicolor*. PSK (polysaccharide K or Krestin) consists of a β-glucan polysaccharide backbone with attached protein. PSP (polysaccharopeptide) is a complex macromolecule consisting of a peptide portion and a polysaccharide β-glucan backbone with various sugar components, such as mannose, xylose, galactose, fructose, arabinose, and rhamnose [61,67]. The primary biological activity of mushroom proteoglycans is determined by their polysaccharide components.

While most mushrooms appear to have immunomodulatory properties due to β-glucans in their cell walls, we focus on well-known examples that have been extensively researched. These include polysaccharides lentinan from shiitake (*Lentinula edodes*) and D-fraction from maitake (*Grifola frondosa*); the proteoglycans PSK (Krestin) and PSP from turkey tail (*Trametes versicolor*); and different polysaccharide fractions from reishi (*Ganoderma lucidum*), chaga (*Inonotus obliquus*), *Agaricus blazei*, and *Cordyceps militaris*.

#### 3.2.2. The Gateway: Uptake and Immune Priming in the GALT

**Figure 4:**
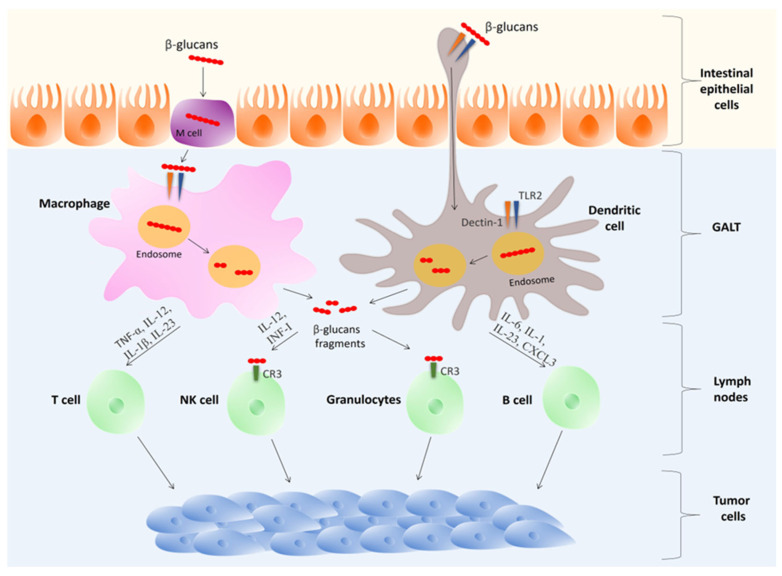
The mechanisms of β-glucans—mediated boosting of the immune system. The detailed explanations are given in the text.

It is widely accepted that ingesting β-glucans stimulates immune cells in the gut-associated lymphoid tissue (GALT) and activates conserved pro-inflammatory pathways. This has been shown to enhance both innate and adaptive immunity [68,69,70]. Consequently, tumor growth is suppressed, and the tumor microenvironment shifts to promote T cell activity [71]. Thus, when combined with adoptive T-cell treatments for cancer, β-glucans serve as accessible, immune-boosting adjuvants [72] (Figure 4).

Humans and animals lack the enzymes necessary to metabolize complex carbohydrates, such as β-glucans. Consequently, when these substances are ingested, they reach the large intestine, where they are metabolized by intestinal flora. A substantial body of research has demonstrated that mushroom polysaccharides have a prebiotic effect and modulate intestinal flora upon reaching the colon. The degradation of these polysaccharides by the microbiota in the digestive tract has been shown to alter the intestinal environment and microbial composition. The reduction in pH induced by polysaccharides promotes the growth and proliferation of beneficial intestinal bacteria and enhances the concentration of total short-chain fatty acids (SCFAs). This, in turn, regulates intestinal homeostasis and the immune response [70,73]. Additionally, β-glucans have been shown to form a gel on the mucosal surface, modulating the resorption of bile salts and altering the intestinal microbiota [60].

In the proximal small intestine, intestinal epithelial cells, also known as pinocytic microfold cells (M cells), carry out phagocytosis of β-glucans (Figure 4). These antigens are then transferred from the intestinal lumen to immune cells in Peyer’s patches, which are part of the gut-associated lymphoid tissue (GALT) [60]. In these patches, β-glucans are ingested by CXCR3^+^ macrophages or CD103+ dendritic cells (DCs). Exposure to β-glucans has been shown to prompt the migration of these cells from the bloodstream to the bone marrow or, via the lymphatic system, to mesenteric or distant lymph nodes [72]. In lymphoid organs, dendritic cells and macrophages fragment and secrete β-glucans into the lymph nodes [74].

The activation of DCs and macrophages is initiated by the presence of β-glucans. These cells then generate various type-I interferons and pro-inflammatory cytokines, which results in augmented differentiation of effector lymphocytes [72]. Additionally, studies have shown that β-glucans can activate dendritic cells that have captured tumor antigens in lymph nodes [68,70]. This process results in the development and activation of antigen-specific CD4^+^ and CD8^+^ T lymphocytes [71]. Furthermore, degraded β-glucan fragments stimulate circulating granulocytes (neutrophils) by binding to the CR3 receptor (CD11b/CD18) and influencing hematopoietic myeloid progenitors in the bone marrow. These events initiate CD3-dependent cellular toxicity in proximity to opsonized (iC3b-coated) neoplastic cells [72]. For example, a study using fluorescein-labeled barley β-glucan detected its fragments in splenic and lymph node macrophages three days after ingestion and in bone marrow macrophages four days after ingestion. This indicates the migration of intestinal immune cells following β-glucan exposure. Granulocytes positive for CR3 and β-(1 → 3)-glucan-fluorescein eliminated iC3b-opsonized tumor cells. This process occurred after the cells were recruited to a region of complement activation analogous to a tumor coated with monoclonal antibodies [69].

#### 3.2.3. Molecular Triggers: Engagement of Pattern Recognition Receptors

**Figure 5:**
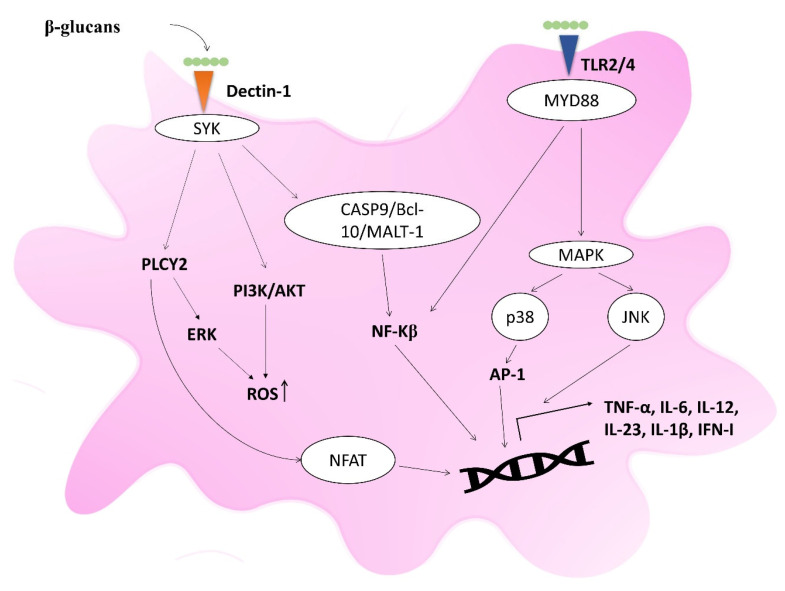
The molecular mechanisms of β-glucan-mediated signaling in immune cells. Detailed explanations are provided in the text.

The immunomodulatory activity of mushroom polysaccharides and proteoglycans begins with their binding to specific pattern recognition receptors (PRRs) on immune cells. This interaction activates downstream signaling cascades, leading to the functional immune outcomes described in the following section (Figure 5).

***Dectin-1 Signaling***. The C-type lectin receptor Dectin-1 is the primary receptor for β-(1 → 3) and (1 → 6)-glucans. Activation of Dectin-1 on myeloid cells (e.g., macrophages, dendritic cells, and neutrophils) triggers Syk-dependent pathways, including CARD9/NF-κB. This leads to phagocytosis and production of pro-inflammatory cytokines, such as TNF-α, IL-6, and IL-12 [75]. This signaling is crucial for bridging innate and adaptive immunity by promoting the differentiation of naïve CD4^+^ T cells into a Th1 or Th17 phenotypes [76]. Examples from medicinal mushrooms include: The β-glucan GFPBW2 from *Grifola frondosa* (maitake) stimulates Dectin-1 in macrophages, inducing Syk and NF-κB activation and enhancing IL-6 and TNF-α production [77]. Similarly, polysaccharides from *Agaricus blazei* and *Cordyceps militaris* also induce Dectin-1 activation [78,79]. Clinically, lentinan from *Lentinula edodes* (shiitake) suppresses intestinal inflammation by directly stimulating Dectin-1 on intestinal epithelial cells and inducing a systemic Th1 response [80,81].

***TLR2/4 Signaling***. Toll-like receptors 2 and 4 (TLR2/TLR4) are key PRRs that detect microbial structures and initiate innate immune responses. Activation of these receptors typically leads to MyD88-dependent signaling, resulting in the activation of NF-κB and MAPK and the production of cytokines such as IL-12, IL-6, and TNF-α [82]. The majority of mushroom-derived polysaccharides and proteoglycans stimulate TLR2/TLR4. The polysaccharide “Cordlan” from *Cordyceps militaris* induces DCs maturation through TLR4-dependent activation of ERK, p38, JNK, and NF-κB [83]. The proteoglycan PSK (Krestin) from Trametes versicolor activates macrophages and induces TNF-α and IL-6 secretion in a TLR4-dependent manner and can also activate NK cells via TLR2 [84,85]. Additionally, polysaccharides from *Armillaria mellea* shift M2 macrophages to an M1 phenotype via TLR2-dependent Akt/NF-κB and MAPK signaling [86], while an *Agaricus blazei* polysaccharide acts as a specific TLR2 agonist to repolarize myeloid-derived suppressor cells (MDSCs) toward an anti-neoplastic M1 state [87].

***The open question of CR3***. A potential supplementary mechanism involves complement receptor 3 (CR3, CD11b/CD18). The hypothesis posits that β-glucan fragments bind to CR3 on neutrophils and natural killer (NK) cells, priming them to destroy tumor cells opsonized with the complement fragment iC3b. This process links innate recognition to complement-mediated cytotoxicity [62,68]. While well-established for yeast- and plant-derived β-glucans [88,89], direct evidence for mushroom-derived β-glucans binding to and activating CR3 remains scarce, with only one early study on lentinan [90]. Consequently, the involvement of CR3 in the anticancer properties of medicinal mushroom polysaccharides is a significant and unresolved knowledge gap that warrants dedicated investigation.

#### 3.2.4. Net Immunological Outcomes: From Molecular Binding to Anti-Tumor Efficacy

The engagement of PRRs (Dectin-1, TLR2/4) by mushroom polysaccharides and proteoglycans initiates a cascade of cellular reprogramming that culminates in a potentiated anti-tumor immune response (Figure 4). The net outcome is the strategic rebalancing of the TME toward effective immune surveillance and away from immunosuppression via several molecular mechanisms. These mechanisms are listed below with certain examples.

***Repolarization of macrophages toward the anticancer M1 phenotype***. Signaling through Dectin-1TLR2/4 reprograms myeloid lineage cells. For example, polysaccharides from *Armillaria mellea* and *Agaricus blazei* transform tumor-promoting M2-like macrophages into tumoricidal M1 macrophages via TLR2/NF-κB/MAPK signaling. This increases the production of IL-12, TNF-α, and nitric oxide [86,87]. Similarly, *Ganoderma lucidum* β-glucans hinder the differentiation and proliferation of myeloid-derived suppressor cells (MDSCs) through the Dectin-1 pathway [91].

***Activation of Innate Effectors***. NK cell activity is enhanced both directly and indirectly. The proteoglycan Krestin (PSK) from *Trametes versicolor* directly activates NK cells via TLR2 [85]. Furthermore, polysaccharides from *Sanghuangporus vaninii* activate DCs and macrophages. This leads to the release of cytokines, such as IL-12 and IFN-γ. These cytokines potently activate NK cells [52].

***Orchestration of Adaptive Immunity***. The maturation and activation of DCs is a pivotal step. Polysaccharide cordlan from *Cordyceps militaris* induces DC maturation via TLR4-dependent activation of NF-κB and MAPK pathways [83]. This DC activation is critical for priming adaptive responses. Importantly, polysaccharides may promote a Th1-dominant response. For example, lentinan from *Lentinula edodes* induces IL-12 production and CD4^+^ Th1 differentiation in the gut [81], whereas polysaccharides from *Grifola frondosa* enhance IL-12 production by DCs to drive an antigen-specific Th1 response [92]. This IFN-γ-rich environment further activates M1 macrophages and drives the proliferation and cytotoxic function of CD8^+^ T cells [52].

***Overcoming Tumor-Induced Suppression***. Immunomodulation governed by mushroom-derived polysaccharides directly counteracts tumor escape mechanisms. Combining *Ganoderma lucidum* β-glucans with gemcitabine decreases PD-L1 expression and increases MHC-II and co-stimulatory molecules on antigen-presenting cells, thereby reversing an immunosuppressive TME [91]. Furthermore, polysaccharides from *Lentinula edodes* (MPSSS) activate the TLR4-NF-κB pathway in cancer-associated fibroblasts (CAFs), thereby eliminating their T-cell inhibitory function [93].

Finally, Table 1 summarizes the primary immunological findings from several preclinical studies on mushroom polysaccharides and proteoglycans.

In summary, the initial molecular interaction of β-glucans with PRRs on immune cells in the GALT translates into a systemic recalibration of immunity. This process amplifies the anti-tumor functions of innate cells (NK cells, M1 macrophages), bridges innate and adaptive immunity via DC activation, and skews the adaptive response toward a durable, cytotoxic Th1/CD8^+^ T-cell axis, thereby creating a hostile microenvironment for tumor growth. Thus, mushroom polysaccharides and proteoglycans, primarily β-glucans, function as potent, orally available immune system modulators. This established immunomodulatory mechanism supports their clinical development not as direct cytotoxic agents, but as well-tolerated immunoadjuvants to be combined with standard cancer therapies.

## 4. The Direct Antineoplastic Activity of Mushroom-Derived Polysaccharides and Proteoglycans

In addition to its impact on the immune system, there are many examples of mushroom-derived polysaccharides that directly confer numerous anticancer properties at the cellular level. According to numerous reports, β-glucans and proteoglycans induce cell cycle arrest, apoptosis, and signaling pathway inhibition.

For instance, *Inonotus obliquus* polysaccharides (IOPS) have been shown to activate AMPK and inhibit glycolysis, oxidative phosphorylation, and ATP production in an AMPK/LKB1-dependent manner [97]. IOPS decreased metalloproteinase expression and inhibited the migration and invasion capabilities of murine melanoma cells. This was associated with the suppression of NF-κB-, c-Jun-, and AKT-dependent signaling pathways [98].

*Agaricus bisporus*-derived polysaccharides were found to downregulate p38 MAPK and the nuclear translocation of p65 NF-κB in murine melanoma cells *in vitro* [99]. The hot water extract of these polysaccharides was cytotoxic to prostate cancer cells, but not to non-cancerous prostate cells. It suppressed DHT-induced PSA expression and the proliferation of prostate cancer cell lines by disrupting androgen receptor signaling through the inhibition of androgen receptor nuclear translocation [100].

*Ganoderma lucidum*-derived polysaccharides suppressed the activity of the ataxia-telangiectasia mutated (ATM) and DNA-dependent protein kinase (DNA-PK) enzymes, thereby sensitizing hepatocellular carcinoma (HCC) cells to radiation [101]. In another study, GL polysaccharides reduced epidermal growth factor receptor (EGFR)/protein kinase B (Akt)-mediated signaling, making tongue cancer cells sensitive to cisplatin. Conversely, GL polysaccharides increased the viability of oral epithelial cells suppressed by cisplatin [102].

Despite the numerous examples of the direct anticancer activity of mushroom-derived polysaccharides and proteoglycans on malignant cells, the physiological importance of this phenomenon remains unclear. First, the molecular mechanisms of β-glucans’ in vitro activity are unknown. Typically, in vitro studies reveal the negative impact of β-glucans on cellular signaling pathways, but they do not provide evidence of how this is achieved. Do cancer cells possess receptors for β-glucans, or are all their effects due to altered osmolarity or the impact of a high concentration of macromolecules on cell membranes? Regarding the latter, we have not found any research in which relevant controls have been used. Typically, authors treat tumor cells with mushroom-derived polysaccharides and compare them with mock-treated cells. In our opinion, the diluent alone is not a relevant control for β-glucans, as they significantly alter osmolarity and may impact cells due to macromolecular crowding, a well-recognized phenomenon. If the direct in vitro effects of β-glucans are driven by either osmolarity or macromolecular crowding, then these effects may be irrelevant in physiological conditions because high concentrations cannot be achieved in vivo.

To overcome this obstacle, we propose the implementation of a more effective macromolecular control. Perhaps this could be a polysaccharide-based component derived from potato or apple starch, for example. We believe such controls will clarify whether mushroom-derived polysaccharides have direct anticancer properties that are not induced by high osmolarity or macromolecular crowding.

Moreover, the intriguing question remains: Are there receptors for mushroom-derived β-glucans and proteoglycans on the surface of tumor cells? Different studies have revealed the presence of Toll-like receptors (TLR2 and TLR4) in epithelial cells, endothelial cells, fibroblasts, and tumor cells of various origins [103,104,105]. The functions of TLR2 and TLR4 in tumor cells are paradoxical and context-dependent; they can either promote tumor growth and survival or lead to cell death and anti-tumor immune responses [103,106,107]. Comprehensive studies are needed to investigate the impact of TLR2/TLR4 expression in malignant cells on the anticancer properties of mushroom-derived β-glucans and proteoglycans and the consequences of their activation.

Beyond this, there is one more serious challenge associated with the direct anticancer activity of mushroom-derived polysaccharides and proteoglycans—their extremely poor absorption. Several studies have shown that small amounts of β-glucan fragments can be found in organs due to engulfment by various intestinal cells, followed by their release [108,109]. However, these extremely low concentrations cannot produce a direct anticancer effect. Additionally, cells release processed fragments of polysaccharides, not the entire polysaccharides.

Given the inability of β-glucans to accumulate in tissues, the physiological importance of their direct anticancer activity is reasonably called into question. For example, they are unable to confer their direct anticancer properties against lung or breast cancer because they cannot come into contact with cancer cells.

**Figure 6:**
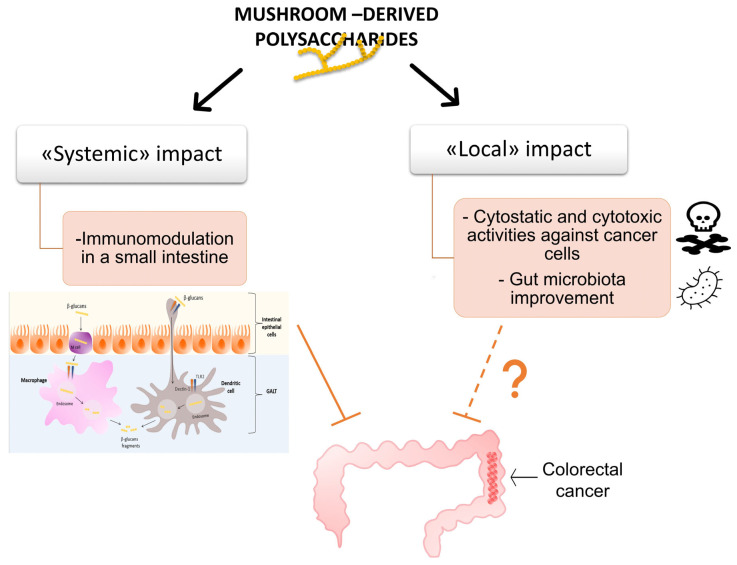
The scheme suggesting that mushroom-derived polysaccharides have both systemic and local impacts on colorectal cancer cells. A solid bar indicates suppression, while a dashed bar indicates presumed suppression of colorectal cancer, as hypothesized in the current review. Detailed explanations are provided in the text.

Therefore, we hypothesized that this could potentially be useful in treating colon cancer (Figure 6). Indeed, polysaccharides and proteoglycans accumulate in large amounts in the intestine after ingestion. In theory, they may exhibit direct anticancer activity against colorectal cancer cells in vivo. In this case, dietary or medical consumption of purified glucans/proteoglycans or entire mushrooms could theoretically be used to prevent and treat colorectal cancer in three ways: by increasing the anticancer immune response, ameliorating intestinal inflammation (colitis), and directly suppressing colorectal cancer cells. Table 2 lists some studies illustrating the usefulness of this approach. Furthermore, we encourage interested readers to consult several relevant reviews [110]. We suggest conducting more studies on the direct and indirect activities of mushroom-derived polysaccharides against *in vitro* and *in vivo* colon cancer models.

## 5. Mushroom-Derived Proteins with Antineoplastic Activities

In addition to polysaccharides and proteoglycans (proteins whose anticancer activity is determined by the polysaccharide part), mushrooms also possess proteins without a polysaccharide part that have antineoplastic properties. Currently, they are generally less significant for cancer therapy than β-glucans and proteoglycans; therefore, we will only briefly discuss them.

The two main categories of anticancer proteins found in mushrooms are fungal immunomodulating proteins (FIPs) and ribosome-inactivating proteins (RIPs) [123].

FIPs have the ability to interact with immune cells and modulate the immune response. These peptides have been tested for their ability to stimulate the production of cytokines by immune cells, as well as for their potential anti-allergy and anti-neoplastic effects. FIPs are grouped into five subgroups based on structure and identity. Fve-type FIPs are the most researched due to their cancer-, immune-, and blood-related properties. These tiny proteins typically have a molecular weight of 13 kDa and 110 to 125 amino acids. The known FIPs are reviewed in [124,125].

FIPs possess immunostimulatory, anti-allergic, and anti-inflammatory activities. Regarding immunomodulation, they balance and enhances the immune response: stimulates cytokine release, activates T- and B-lymphocytes, inhibits histamine release from mast cells [125,126,127].

Among the known FIPs, LZ-8 and GMI are the most well-characterized. They are found in *Ganoderma lucidum* (reishi or lingzhi) and *Ganoderma microsporum*, respectively, and they share structural and sequence similarities [126]. In addition to their role in immunomodulation, LZ-8 and GMI directly affect different features in cancer cells, making them promising candidates for study as potential adjuvants (Table 3).

Recently, GMI (Reishimmune-S) was studied in two clinical trials as an adjuvant treatment for patients with breast and head and neck cancers. Reishimmune-S treatment improved cognitive function, tiredness, and sleeplessness in breast cancer patients while altering the composition of circulating immune cells [139]. In the second trial, the Reishimmune-S supplement significantly reduced chemotherapy-induced oral mucositis [140]. Based on the history of GMI research, the GMI database has been developed: https://gmibio.com/.

Taken together, the aforementioned results of clinical trials and the preclinical data listed in Table 3 suggest the potential effectiveness of FIPs as adjuvants in cancer therapy. Further preclinical and clinical studies addressing their efficacy and safety are highly recommended.

Another class of mushroom-derived proteins that have direct anticancer activity are ribosome-inactivating proteins (RIPs). These enzymes inhibit protein synthesis by damaging ribosomes. Examples of RIPs found in mushrooms include edodin from shiitake (*Lentinula edodes*) [141], marmorin from *Hypsizigus marmoreus* [142], and volvarin from *Volvariella volvacea* [143]. These proteins have ribosomal RNA (rRNA) N-glycosylase activity, which leads to cell death. They also possess cytotoxic properties and show potential for biomedical and biotechnological applications.

Although preclinical and early clinical data on FIPs are promising, they have not yet been widely accepted as a new class of pharmaceutical drugs. Most of the evidence comes from laboratory and animal studies, and more human clinical trials are needed to confirm their efficacy and safety. FIPs are indeed able to provide balanced, multi-targeted immune modulation. Despite their promise, large-scale production and effective delivery of these protein-based drugs, as well as bioavailability, are major challenges for FIPs. Unlike small molecules, FIPs can be broken down in the digestive system and may not easily enter cells or the bloodstream when taken orally [127]. Current research is actively addressing this issue by using recombinant production to engineer microorganisms [144] and develop advanced delivery systems, such as encapsulation, to protect FIPs and enhance their absorption and stability [127,145,146].

## 6. Potential Low-Molecular-Weight Compounds from Mushrooms

In addition to polysaccharides and proteoglycans, which significantly boost anticancer immunity, mushrooms contain secondary metabolites with various biological activities that directly affect cancer cells by mostly inhibiting key signaling pathways.

The development and progression of cancer are driven by the dysregulation of core cellular signaling pathways that govern proliferation, survival, and metabolism [147]. Genomic analyses of human tumors reveal frequent alterations in key pathways, making them prime targets for therapeutic intervention. The most commonly altered pathways include the PI3K/AKT/mTOR and MAPK (RTK/RAS), which promote oncogenic growth and survival; the Wnt/β-catenin, which regulates stemness and proliferation; the p53, which mediates genomic integrity and apoptosis; and the unfolded protein response (UPR), which manages endoplasmic reticulum stress and influences cell fate [148]. For more information on cancer signaling, please refer to the following reviews: [149,150,151].

**Figure 7:**
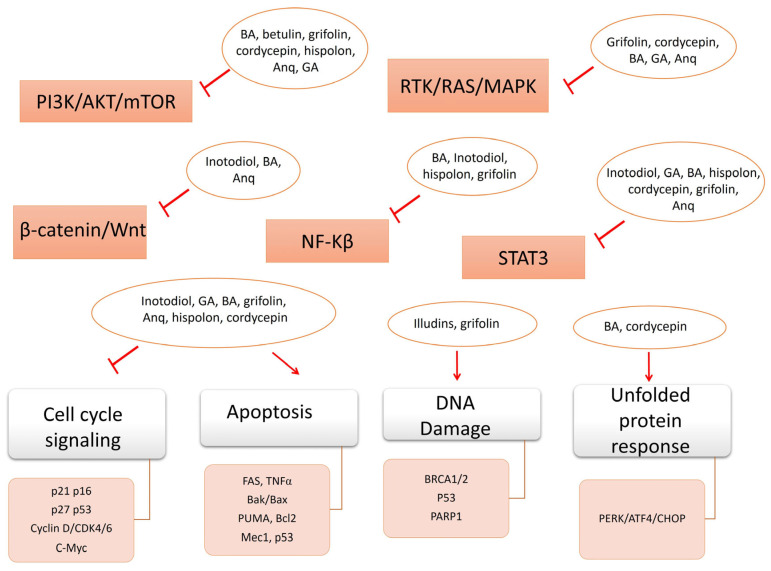
Promising low-molecular weight compounds from mushrooms target key signaling pathways in cancer. The description is given in the text. Abbreviations: BA—betulinic acid, GA—ganoderic acid, Anq—antroquinonol.

The aforementioned pathways represent critical nodes that are often modulated by bioactive compounds, including those derived from mushrooms, in order to exert anticancer effects. Figure 7 summarizes the effects of certain low-molecular-weight compounds on common signaling pathways and their components, which are usually deregulated in different malignancies.

Below, we discuss several subclasses of low-molecular-weight compounds from mushrooms. In this chapter, we focus on mushroom-derived compounds with robust, multifaceted evidence of anticancer activity, including well-characterized molecular mechanisms and demonstrated selectivity between cancerous and noncancerous cells.

### 6.1. Terpenes

Although many mushroom species produce terpenes, three of the most well-known examples with potential medicinal applications are *Inonotus obliquus* (chaga), *Ganoderma lucidum* (reishi), and *Albatrellus confluens*. We discuss these species in detail below.

#### 6.1.1. Triterpenoides

Chaga (*Inonotus obliquus*) is well-known for its medicinal properties in northern regions where it is widely grown, including Russia, Eastern Europe, and Canada [152]. It is an exceptional mushroom, and the biological properties of its low-molecular-weight compounds are studied more thoroughly than polysaccharides. First, chaga is rich in biologically active terpenoids. In Russia, this has led to a specific medical application of Chaga: a “double extraction” procedure that combines polysaccharides and low-molecular-weight constituents to maximize the mushroom’s health benefits. This process typically involves extracting chaga with 80% ethanol, followed by extraction with boiling water. In the first step, low-molecular-weight compounds, including lanostane triterpenoids, are extracted. Then, in the second step, water-soluble polysaccharides are extracted using boiling water. Finally, the two extracts are combined.

The main triterpenoids found in chaga are inotodiol, trametenolic acid, betulinic acid, and betulin [153,154]. Other minor terpenes with antineoplastic properties include inonotixides A and B [155].

In a study by Wang et al., the following triterpenoids were identified as primary constituents of Chaga-derived methanolic extract: inotodiol, trametenolic acid, 3-hydroxy-lanosta-8,24-dien-21-al, and betulinic acid [156]. The extract was shown to induce cell cycle arrest in the G0/G1 phase by decreasing the protein levels of cyclin D1, CDK4, cyclin E, and p-Rb. Furthermore, the extract demonstrated a synergistic effect when combined with cisplatin and trastuzumab, resulting in the downregulation of HER2 activation in a HER2-overexpressed breast cancer cell model [156]. Interestingly, dihydrofolate reductase (DHFR), a pivotal enzyme in one-carbon metabolism, was identified as a direct target of betulinic acid, which significantly suppresses its enzymatic activity.

Other researchers examined the *Inonotus obliquus* extract and identified 13 lanostane triterpenoids. Of these, inonostutriol E demonstrated optimal cytotoxicity against various cancer cell models. The present study demonstrated an association between suppression of the JAK2/STAT3 signaling pathway and occurrence of the aforementioned phenomenon [157].

Other studies have identified several chaga-derived lanostane triperpenoids: 3β-hydroxylanosta-8,24-dien-21-al and 3β-hydroxy-5α-lanosta-8,25-dien-21-oic acid [158], inonotsutriols A and E, and inonotsutriol D, as well as 3β,22α-dihydroxylanosta-8,25-diene-24-one [155]. These substances possess cytotoxic activity against cancer cell models. However, the precise mechanism through which they exert their anticancer activities remains unclear.

**Figure 8:**
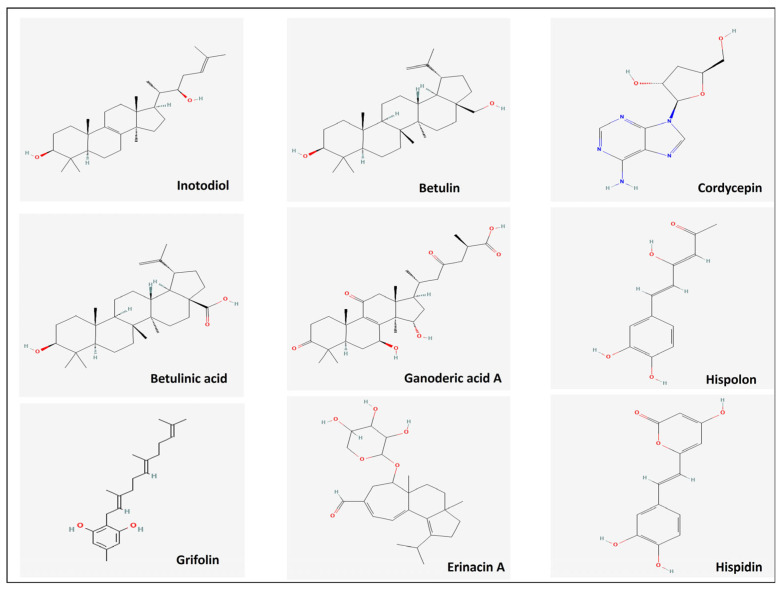
The molecular structures of selected well-known, mushroom-derived, low-molecular-weight compounds with antineoplastic properties.

***Inotodiol*** (22R)-lanosta-8,24-diene-3β,22-diol and ***trametenolic acid*** (3β-hydroxylanosta-8,24-dien-21-oic acid) are evidently the primary triterpenoids in Chaga, based on their quantitative content (Figure 8). The antitumor effect of inotodiol was initially demonstrated using a mouse skin carcinogenesis test [159], which showed a significant reduction in papilloma formation. Additionally, intraperitoneal administration of inotodiol has been shown to extend the survival of mice with leukemia [160]. Inotodiol was found to inhibit the cell cycle and affect the expression of cyclin E and p27. Furthermore, it induced DNA fragmentation and apoptosis [154,161,162].

In a study by Zhang et al., a diabetic rat model with induced breast cancer was used to determine the effects of inotodiol. The study’s findings demonstrated that inotodiol significantly reduced proliferating cell nuclear antigen (PCNA) positivity and increased the number of apoptotic cells in breast cancer tissues [163]. At the molecular level, this was associated with decreased expression of β-catenin and its transcription targets, c-Myc and Cyclin D1. The antitumor effect of inotodiol was also associated with reduced blood glucose, cholesterol, triglyceride, and high-density lipoprotein levels and improved glucose tolerance [163]. In a separate study, the inotodiol- and trametenolic acid-enriched fractions of a Chaga-derived ethanolic extract were found to significantly reduce tumor volume in a 4T1 breast cancer mouse model. The authors demonstrated that these fractions significantly suppress mTOR signaling and induce AMPK-dependent autophagy without compromising the cytotoxic effects of conventional drugs [164].

A pharmacokinetic study of inotodiol in mice revealed that after an intravenous dose of 2 mg/kg, the terminal half-life (T_1/2_) was 49.35 min. In contrast, an oral dose resulted in an extremely low absolute oral bioavailability of only 0.45% [165]. This low bioavailability is due to inotodiol’s poor aqueous solubility and low intestinal absorption, common issues for nonpolar sterols. Researchers have developed advanced delivery systems to improve its absorption. One such system, a microemulsion formulation, increased the oral bioavailability of inotodiol to 41.32% at a dose of 4.5 mg/kg in mice [166]. An inclusion complex with γ-cyclodextrin increased the relative AUC by 4.96-fold compared to unformulated inotodiol [167].

Unlike inotodiol, there is limited data on the biological activity of trametenolic acid. Studies have shown that trametenolic acid is less cytotoxic against cancer cells than inotodiol and betulinic acid. It has been demonstrated that trametenolic acid regulates RhoC (Ras homolog gene family, member C) in the HepG2 hepatoma cell line. It also inhibits the RhoC/ROCK1/MMP2/MMP9 signaling pathway, impeding tumor growth in vivo [168]. Notably, trametenolic acid reversed P-proteoglycan-mediated multidrug resistance in triple-negative breast cancer by downregulating P-gp expression [169].

***Betulinic acid*** (BA) is a pentacyclic lupane-type triterpenoid produced by birch trees (*Betula* sp.) and stored by the Chaga mushroom (Figure 8), a parasite of birch trees. Several research groups have reported that BA suppresses various signaling pathways, including PI3K/AKT/mTOR, AK/STAT, VEGF, and EGFR. These groups have also reported the induction of autophagy, cell cycle arrest, and apoptosis [170]. BA down-regulates PI3K-110α, PI3K-p85, p-AKT (S473), and p-AKT (T308) in a cervical cancer cell line [171]. In the context of hepatocellular carcinoma, BA significantly suppresses PI3K/AKT/mTOR and induces autophagy [172].

BA has been shown to repress Sp1, Sp3, and Sp4 transcription factors, thereby reducing the levels of EGFR by suppressing their interaction with the EGFR promoter [173]. Additionally, BA has been shown to increase the radiosensitivity of oral squamous cell carcinoma by inducing PTEN [174].

Consistent with these findings, BA has been demonstrated to suppress pancreatic CSCs by upregulating AMPK. Notably, BA reduced the expression of three pluripotency factors (Oct4, Sox2, and Nanog) and some EMT markers. AMPK knockdown effectively counteracts the effects of BA on EMT and stemness [175].

In the context of insulin-resistant HepG2 hepatocellular carcinoma cells, BA has been observed to impede the CAMKK2-AMPK-SREBP1 signaling pathway. This leads to a decrease in SREBP1 mRNA expression, which then prevents it from translocation into the nucleus. Concurrently, this process led to the suppression of the mammalian target of rapamycin (mTOR) and S6 kinase (S6K). Furthermore, it resulted in decreased intracellular lipid accumulation [176].

Separate research on insulin-resistant HepG2 hepatocellular carcinoma cells predicted that BA would bind directly to the insulin receptor (IR) or the insulin-like growth factor 1 receptor (IGF1R). BA was found to suppress insulin and IGF1 signaling by inhibiting the post-translational modification of the IRS1/PI3K/AKT-pT308 and IGF1/mTORC2/AKT-pS473 pathways. Consequently, the mTOR/S6K/S6 pathway was inhibited and de novo lipogenesis decreased [177].

Several research groups have demonstrated that the presence of BA inhibits JAK/STAT3 signaling in both *in vitro* and *in vivo* cancer models [178,179,180]. Furthermore, it has been demonstrated that BA exerts an anti-proliferative effect on prostate cancer cells by modulating VEGF expression. This is accomplished by preventing HIF-1α and STAT3 from binding to the VEGF promoter, which impedes angiogenesis [180].

Glucose-regulated protein 78 (GRP78) has recently been identified as a direct binding partner of BA. The authors demonstrated that BA induces GRP78-mediated endoplasmic reticulum (ER) stress in breast cancer through activation of the PERK/eIF2α/CHOP signaling pathway, thereby sensitizing breast cancer cells to taxol. It is important to note that normal mammary epithelial cells (MCF10 cell line) exhibited significantly reduced sensitivity to BA [181].

In a separate study, the same authors demonstrated that BA-induced endoplasmic reticulum (ER) stress resulted in GRP78/PERK-mediated β-catenin downregulation. This led to a decrease in c-Myc and the inhibition of glycolysis and respiration. This effect was accompanied by an increase in E-cadherin and a suppression of vimentin, MMP2, and MMP9. Furthermore, the effects of BA have been corroborated in an *in vivo* breast cancer model [182].

Regarding the inhibition of metabolic reprogramming in cancer, BA has been demonstrated to inhibit glycolysis and respiration in melanoma and breast cancer cells [183,184]. Consistent with this evidence, BA significantly reduced levels of c-Myc, LDHA, and PDK1. In contrast to its effects on cancer cells, BA exhibits no embryotoxic or teratogenic effects during the development of zebrafish embryos. Interestingly, BA has been observed to upregulate glycolysis in non-cancerous cell lines without negatively affecting their survival [185].

BA has low oral bioavailability (less than 1%) due to its poor water solubility [186]. After injection in mice, its half-life is moderately long (approximately 11.5 h), and it distributes widely into tissues such as fat and the spleen [187]. Advanced formulations like self-nanoemulsifying drug delivery systems (SNEDDS) can increase its absorption in preclinical models by 3.9- to 17-fold [188].

***Betulin*** is a lupane-type pentacyclic triterpene (Figure 8) abundant in birch bark and also accumulated by chaga. Numerous studies have demonstrated that betulin induces G0/G1 cell cycle arrest, autophagy, and activation of the intrinsic (mitochondrial) apoptotic pathway in different cancer models (reviewed in [189]).

In colorectal cancer cell and mouse models, betulin mitigated the activation of the PI3K/AKT/mTOR pathway, inducing autophagy, cell cycle arrest, and apoptosis. Notably, betulin was found to significantly reduce lung metastasis [190].

In multidrug-resistant human renal carcinoma cells, betulin reduces multidrug resistance protein 1 (MDR-1) expression, increasing sensitivity to etoposide [191]. In several other renal carcinoma cell lines, betulin suppressed glucose consumption and lactate production, inhibited the mTOR signaling pathway, and down-regulated HK2 and PKM2, two rate-limiting enzymes in glycolysis [192]. Furthermore, betulin has been shown to compromise mTOR activity and induce autophagy in osteosarcoma cells [193].

Betulin is a known SREBP1 inhibitor that interferes with its processing [194]. It suppresses the SREBP pathway, decreases cholesterol and fatty acid biosynthesis, and reduces atherosclerotic plaques, thereby improving insulin sensitivity. According to the aforementioned evidence, betulin exhibits a synergistic effect with sorafenib, suppressing hepatocellular carcinoma by downregulating genes that encode enzymes involved in *de novo* lipogenesis (FASN, ACC, and ACLY). Notably, betulin significantly reduced the volume of hepatocellular carcinoma xenografts when administered with sorafenib [194].

However, betulin has very poor oral bioavailability in its native form due to its low aqueous solubility (approximately 0.08 µg/mL) and highly hydrophobic nature [189,195]. Researchers are focusing on overcoming this barrier with advanced drug delivery systems. For instance, forming nanoparticles increased betulin’s bioavailability by 1.21 times [196], while cyclodextrin complexes enhanced solubility and showed improved anti-tumor and hepatoprotective effects *in vivo* [197,198]. Studies confirm that betulin has low systemic toxicity [195].

***Ganoderma lucidum***, also known as reishi, is one of the most well-known edible mushrooms, with a history of medicinal use dating back more than 2000 years. Its main immune-boosting and antitumor effects come from polysaccharides, but it also contains triterpenes with antineoplastic properties [22].

Several studies have identified the anticancer properties of terpenes derived from methanolic and ethanolic extracts of *Ganoderma* species. These extracts have been shown to downregulate the expression of various proteins in several cancer cell models. These include VEGF, EGFR, p38 MAPK, HER2/PI3K/Akt, and Ras/Raf/MEK/ERK [199,200,201,202,203]. A total of 495 triterpenoids have been identified in 25 different *Ganoderma* species [204].

Liang et al. summarize the molecular mechanisms underlying the biological activities of the seven major ganoderic acids isolated from *Ganoderma lucidum*: A, C2, D, F, DM, X, and Y [205]. These compounds exhibit significant pharmacological effects, particularly anticancer and anti-inflammatory activities, by modulating signaling pathways such as NF-κB, AP-1, p53, and caspases, leading to cell growth inhibition, induction of apoptosis, promotion of autophagy, and suppression of metastasis and angiogenesis. Among these triterpenoids, ganoderic acid D is believed to exhibit higher toxicity, whereas ganoderic acid A (Figure 8) is the most extensively studied compound from *Ganoderma* sp. [206].

***Ganoderic acid A*** (GAA) has been demonstrated to exert a number of significant effects in models of breast cancer cells. These effects include downregulating JAK2 and STAT3 activation, upregulating ROS production, and inducing cell cycle arrest and apoptosis [207].

Recent research has identified GAA as a compound that targets E3 ubiquitin ligase MDM2, which is a key negative regulator of the tumor suppressor protein p53 [208]. MDM2 inhibitors have the potential to disrupt the MDM2-p53 protein–protein interaction, thereby stabilizing p53. They facilitate the restoration of p53 activity in tumors that express wild-type p53. However, our recent findings have demonstrated that GAA cannot efficiently target MDM2 or stabilize p53 in non-small cell lung cancer (NSCLC) models, unlike nutlin 3a (a well-known synthetic MDM2 inhibitor) and berberine (a natural compound derived from plants) [209]. Nevertheless, according to the existing literature, GAA derivatives that efficiently target MDM2 have been discovered [208,210].

Chemotherapy-related fatigue is a debilitating symptom that significantly impacts the quality of life of cancer patients by interfering with their physical and social functioning. This condition involves dysfunction in the muscular and central nervous systems. In mice bearing colon tumors, GAA modestly enhanced the activity of 5-FU. However, this combination significantly reduced chemotherapy-induced fatigue, enhancing muscle quality and mitochondrial metabolic functions. Additionally, it reduced inflammation in the central nervous system [211].

The scientific literature provides comparatively little information on other types of ganoderic acids. For example, ganoderic acid X has been shown to inhibit topoisomerase in hepatocellular carcinoma cells, thereby inducing apoptosis [212]. Both GAA and ganoderic acid DM were found to suppress the expression of several oncogenes, including c-Myc, β-catenin, vascular endothelial growth factor (VEGF), phospho-AKT (S473T), and Mcl-1, in meningioma cell lines [213]. Ganoderic acid D (GAD) induces autophagy-mediated cell death and suppresses AKT/mTOR signaling in esophageal squamous cell carcinoma models [214].

Notably, two research groups have demonstrated the negative effects of GAD on metabolic reprogramming. GAD has been shown to suppress glucose uptake and the production of lactate, pyruvate, and acetyl-coenzyme A in colon cancer cells. This process is facilitated by SIRT3 up-regulation [215]. In a separate study, GAD was found to reverse gemcitabine susceptibility in triple-negative breast cancer cells. This reversal was achieved by inducing the proteasomal degradation of HIF1α, resulting in the downregulation of its transcriptional targets that code for GLUT1, HK2, and PKM2 [216].

Recent studies have suggested that ganoderic acids’ anticancer properties may extend beyond their direct impact on tumor cells. In a collaborative study, Song et al. demonstrated that administering GAA to a mouse model of colon cancer enhances the anticancer effect of oxaliplatin by increasing T cell toxicity [217]. In a separate study, galectin-1 (Gal-1) was identified as a direct target for ganoderic acid T (GAT). Gal-1 is a pivotal molecule that plays a critical role in immunosuppression in various types of neoplasia. The authors demonstrated that GAT induces Gal-1 ubiquitination and downregulation, thereby modulating the microenvironment and enhancing the efficacy of chemotherapy and immunotherapy in mouse models of ovarian and breast cancer [218].

Ganoderic acids are characterized by rapid absorption; however, their oral bioavailability is generally low and variable due to poor solubility [219,220]. The bioavailability of specific compounds, such as GAA, is only 10.38–17.97% in rats [221]. However, advanced formulations, such as solid lipid nanoparticles, can dramatically enhance absorption. For example, the bioavailability of GAD increased from 22% to 70% with these formulations [222,223]. Despite their rapid absorption, these compounds typically have short half-lives (0.4–2.5 h) and their overall pharmacokinetics can be significantly impacted by factors such as food intake [219,221,223].

#### 6.1.2. Diterpenes

The fungus *Hericium erinaceus*, also known as the lion’s mane mushroom, is the source of the ***erinacines*** (erinacines A–I), which are a class of cyathane diterpenes with angularly fused 5/6/7 tricyclic cores. These compounds exhibit various pharmacological activities. Erinacine A (Figure 8) is the most prevalent of the various identified erinacines and the subject of the most extensive research [224]. It has been demonstrated to possess several anticancer properties, particularly affecting signaling pathways (reviewed in [225]).

An unusual molecular mechanism for the anticancer activity of erinacine A has been proposed by one research group. Rather than inhibiting AKT/mTOR, erinacine A activates this critical signaling pathway to induce apoptosis in cell and animal models. First, it was demonstrated that erinacine A can suppress the growth and migration of colon cancer cells. Furthermore, it was discovered that it significantly reduced xenograft growth at low concentrations (1–5 mg/kg) while simultaneously activating the AKT/mTOR signaling pathway in *in vitro* and *in vivo* models. The authors hypothesized that erinacine A-mediated reactive oxygen species (ROS) production activates the AKT/mTOR axis, which then upregulates the activity and expression of ROCK1/LIMK2/cofilin, resulting in antineoplastic activity [226].

In a subsequent study by the same research group, it was discovered that erinacine A induces both the intrinsic and extrinsic pathways of apoptosis in gastric cancer cells. This was associated with strong activation of the FAK/AKT/p70S6K/PAK1 axis and increased expression of the 14-3-3 sigma protein (1433S) and microtubule-associated tumor suppressor candidate 2 (MTUS2). Interestingly, the use of rapamycin (an mTOR inhibitor) and Y15 (an FAK inhibitor) resulted in the abolition of erinacine A-mediated apoptosis and 14-3-3 sigma/MTUS2 expression [227].

Further research revealed that erinacine A-induced extrinsic pathway apoptosis in colorectal cancer cells and xenografts is associated with Jun N-terminal kinase (JNK1/2) activation, as well as NFκB p50 and p300 (JNK/p300/p50) activation and histone H3 acetylation (H3K9K14Ac) of TNFR, Fas, and FasL promoters. Notably, the use of SP600125, C646, and PDTC—which inhibit JNK, p300, and NFκB activation, respectively—resulted in the suppression of erinacine A-induced cell death and the suppression of promoter acetylation of Fas, FasL, and TNFR on histone 3 at K9 and K14 residues [228].

Finally, the authors demonstrated that another erinacine, erinacine S, can induce extrinsic apoptotic pathways in gastric cancer cells and xenografts. This is accomplished through the strong upregulation of the AKT/FAK/PAK1 axis and the transcriptional activation of FasL and TRAIL via H3K4 trimethylation on their promoters [229].

While intriguing, the manner in which erinacine A activates the AKT/mTOR signaling pathway is debated in regard to its anticancer effects. Further studies are necessary to elucidate the molecular mechanisms by which erinacine A mediates its antineoplastic properties in various malignancies.

Preclinical studies in rats indicate that the erinacines A and S have moderate oral bioavailability, at approximately 24% and 15%, respectively [230]. Erinacine A was detected in the brain one hour after oral administration in rats, reaching a peak concentration at 8 h [230]. Quantitative data from rats shows that the plasma T(max) of ercinacine S is approximately 4.5 h, and the T_1/2_ (half-life) is about 7.3 h [231]. Both compounds are primarily eliminated through the feces [230,231].

#### 6.1.3. Sesquiterpenes

***Grifolin***, (5-methyl-2-[(2E,6E)-3,7,11-trimethyldodeca-2,6,10-trienyl] benzene-1,3-diois a sesquiterpene with an aromatic alcoholic core (Figure 8). It is the primary antineoplastic compound found in *Albatrellus* species [232]. It exhibits antibacterial, antifungal, antileishmanial, antioxidant, and antineoplastic activities. Various studies have demonstrated grifolin-mediated G1 cell cycle arrest and induction of the intrinsic pathway of apoptosis in different cancer cell models [233].

First of all, grifolin was shown to directly bind to ERK1/2, thereby impeding the activation of this signaling pathway in cancer cells [234]. Second, computer simulations indicated that grifolin may bind to PI3KCA (phosphatidylinositol-4,5-bisphosphate 3-kinase catalytic subunit alpha) [235]. Several studies have demonstrated grifolin-induced inhibition of the PI3K/AKT/mTOR signaling pathway in various tumor cell models [235,236,237,238]. Therefore, grifolin appears to possess the capacity to impede two primary oncogenic signaling pathways: PI3K/AKT and ERK/MAPK.

Another important antineoplastic activity of grifolin is its negative impact on DNMT1 (DNA methyltransferase 1), a key enzyme involved in DNA methylation and important for maintaining methylation patterns. DNMT1 is crucial for tumor growth and recurrence, and it is highly expressed in cancer stem cells, resulting in the silencing of tumor suppressor genes due to hypermethylation of their promoters [239]. *In vivo* studies have shown that grifolin downregulates DNMT1 levels and reactivates the expression of several tumor suppressor genes, including p16, PTEN, and DAPK1 (death-associated protein kinase 1) [234,240]

The Epstein–Barr virus-encoded latent membrane protein 1 (EBV-LMP1) is linked to nasopharyngeal carcinoma pathogenesis by triggering cell signaling pathways that promote transformation, proliferation, immune escape, invasiveness, epigenetic modification, and metabolic reprogramming [241]. Luo et al. demonstrated that LMP1 upregulates DNMT1 expression and activity, leading to metabolic reprogramming in nasopharyngeal carcinoma. Grifolin, in turn, can attenuate glycolytic flux and restore mitochondrial OXPHOS function by inhibiting DNMT1 expression and activity [242].

The DNMT inhibitors used in clinics, 5-aza-2′-deoxycytidine and 5-fluoro-2′-deoxycytidine, are toxic. Grifolin, on the other hand, has low toxicity [233,243]. Its water-miscible prodrug, PEG5-grifolin, exhibits superior demethylation activity compared to 5-aza-2′-deoxycytidine in preclinical studies and is highly soluble [240]. Therefore, grifolin is a promising agent for downregulating DNMT1.

Grifolin is a compound of significant pharmacological interest, yet we could not find basic pharmacokinetic data on it. Grifolin is poorly soluble and degrades under mild conditions. PEG5-grifolin increased the solubility by over 1000-fold compared to the parent compound, and it has a half-life of approximately 87 days at 25 °C in PBS [240].

### 6.2. Phenolics and Other Compounds

Phenolic compounds, predominantly phenolic acids (e.g., caffeic, p-coumaric, gallic, cinnamic, chlorogenic, and syringic acids) and flavonoids (e.g., myricetin, rutin, naringenin, and quercetin), are minor metabolites that are present in all mushrooms. Since these compounds are not primarily responsible for the anticancer activity of mushrooms, they will not be discussed in detail in this review. However, they may contribute to the resulting anticancer activity, as all of these compounds possess antineoplastic properties. Furthermore, phenolic compounds are typically responsible for antioxidant capacity. Readers interested in this topic are referred to excellent reviews and experimental papers on the content of phenolic compounds in mushrooms and their anticancer properties [244,245,246,247,248].

***Antroquinonol***, a naturally occurring phenolic compound derived from the medicinal fungus *Antrodia camphorata*, has received growing attention due to its reported biological activities, including potential anticancer effects [249]. Although research on this molecule is still in its early stages, several preclinical studies have begun evaluating its therapeutic relevance using animal models.

Antroquinonol exhibits significant cytotoxic effects against various human cancer cell lines, including lung, liver, and leukemia cells, with IC_50_ values in the low micromolar range. These effects are independent of Ras mutational status. Mechanistic studies revealed that antroquinonol directly binds to and inhibits protein isoprenyltransferases, specifically farnesyltransferase (FTase) and geranylgeranyltransferase I (GGTase I). This inhibition blocks the post-translational prenylation of Ras and Rho family proteins, resulting in the accumulation of their inactive forms and subsequent disruption of oncogenic signaling. Suppressing these pathways ultimately leads to autophagy-associated cancer cell death [250].

Additionally, antroquinonol has been shown to inhibit the proliferation of hepatocellular carcinoma cells and induce G1-phase cell-cycle arrest, followed by apoptosis. These effects are associated with the activation of AMP-activated protein kinase (AMPK) and the inhibition of the mTOR signaling pathway. This is evidenced by the decreased phosphorylation of mTOR, p70S6K, and 4E-BP1. This suppresses protein synthesis and tumor cell growth [251].

More recently, antroquinonol was reported to suppress cancer stem cell–like properties in colon cancer models. The compound significantly reduced tumor sphere formation, migration, and invasion while downregulating genes related to pluripotency and stemness. These effects were mediated by inhibiting the PI3K/AKT/β-catenin signaling axis. These findings highlight the potential of antroquinonol as a cytotoxic agent and a promising compound for preventing tumor progression, metastasis, and recurrence [252].

*In vivo* evidence further supports its anticancer efficacy. Using a C6 rat glioma xenograft model, Thiyagarajan et al. demonstrated that antroquinonol significantly reduced tumor volume without affecting body weight, indicating good tolerability at the tested doses. Histological examination revealed extensive tumor cell death. Major organs, such as the liver, kidneys, and spleen, showed no detectable pathology, and biochemical markers (creatinine, ALT, and AST) remained within normal ranges. Importantly, antroquinonol decreased the protein levels of Src, pSrc, FAK, pFAK, Rac1, and cdc42. Together, these findings suggest that antroquinonol exerts a potent tumor-suppressive effect *in vivo* while maintaining a favorable safety profile, reinforcing its promise as a therapeutic lead compound derived from medicinal mushrooms [253].

Based on these preclinical observations, antroquinonol has advanced to the early stages of clinical evaluation. In an open-label, first-in-human phase I trial conducted by Lee et al., the compound was administered to patients with metastatic non-small-cell lung cancer that had progressed despite having received at least two prior systemic therapies. The study primarily assessed dose-limiting toxicities, pharmacokinetics, and safety across escalating oral doses ranging from 50 to 600 mg per day. Notably, no dose-limiting toxicities were observed at any dose level, and the maximum tolerated dose was not reached, suggesting a favorable safety margin. The most common adverse events were mild gastrointestinal symptoms. There were no reports of treatment-related mortality or grade 4 toxicities [254]. Although efficacy was not the primary endpoint, clinical findings suggested early signs of disease stabilization. Several patients achieved stable disease during treatment. Pharmacokinetic assessments revealed rapid absorption and elimination with dose-proportional increases in exposure under multiple-dose conditions. Overall, these results support the translational potential of antroquinonol and justify further investigation in larger, controlled Phase II trials [254].

Another study, a phase I/II trial, demonstrated the feasibility of combining antroquinonol with gemcitabine and nab-paclitaxel (Gem/Nab-P) as a first-line strategy for metastatic pancreatic cancer. The study also determined the maximum tolerated dose of antroquinonol to be 300 mg three times a day (TID). This regimen showed promising efficacy, with median progression-free and overall survival of 5.3 and 12.6 months, respectively. Furthermore, the combination resulted in fewer hematological and non-hematological adverse events than Gem/Nab-P alone, though it caused manageable gastrointestinal discomfort [255].

Regarding its pharmacokinetics in humans, antroquinonol is quickly absorbed, reaching maximum plasma concentration (T_max_) in 1.00 to 4.05 h. It is also eliminated fairly rapidly, with an elimination half-life ranging from 1.30 to 4.33 h. This half-life appears to be independent of the administered dose [254].

In its natural form, antroquinonol has poor aqueous solubility (less than 0.1 mg/mL), which limits its oral bioavailability and poses a challenge to its therapeutic application. Advanced formulations, such as chitosan-silicate nanoparticles, have been developed to overcome this limitation. These formulations significantly improve the delivery and absorption of antroquinonol in animal models [256].

***Hispolon*** (6-(3,4-dihydroxyphenyl)-4-hydroxy-6,8-dihydro-5H-benzo [1,2-b:4,5-b′]dithiophene-2-one) (Figure 8) is considered the primary biologically active, low-molecular-weight constituent of the traditional medicinal mushrooms *Phellinus linteus* (Sanghuang), *Phellinus ignarius*, and *Phellinus lonicerinus*. Hispolon confers anti-cancer, anti-inflammatory, anti-diabetic, and immunomodulatory properties [257]. Various studies have demonstrated hispolon-mediated cell cycle arrest, apoptosis, and autophagy induction in different cancer models [258]. For example, hispolon induced G2/M cell cycle arrest and apoptosis and significantly reduced the volume of glioblastoma xenografts in a murine model at a dose of 5 mg/kg [259] (Table 3).

Depending on the cellular genetic background, it seems that the activation of either ERK, JNK1/2, or p38 MAPK is required for hispolon-induced apoptosis. In AML cell lines, for example, inhibiting JNK1/2 kinases significantly reduced hispolon-mediated apoptosis and the activation of caspase-8, -9, and -3. In the same study, hispolon significantly reduced the growth of AML xenografts in mice [260]. In another study examining oral squamous cell carcinoma, only JNK1/2 activation, but not ERK or p38 MAPK activation, was necessary for hispolon-induced apoptosis [261].

Several studies demonstrate that hispolon suppresses AKT/mTOR signaling and induces autophagy in various cancer models [262,263,264]. In prostate cancer cells, hispolon suppresses STAT3 phosphorylation [265]. In a murine melanoma cell model, hispolon was shown to suppress the activities of mitochondrial respiratory complexes I and IV [266].

One study has shown that hispolon induces MDM2 degradation through the lysosomal pathway [267]. In another paper, the authors showed that hispolon induces MDM2 ubiquitinylation and degradation regardless of p53 status via activation of the ERK signaling pathway [268]. Interestingly, two other studies have also shown that hispolon induces ERK activation, which is necessary for hispolon-mediated cell cycle arrest and apoptosis in a hepatoma cell model [269], as well as for the induction of autophagy and the suppression of cervical cancer cell motility [263].

Using a bioinformatics approach, Li et al. identified the following targets for hispolon in TNBC cancer: epidermal growth factor receptor (EGFR), insulin-like growth factor binding protein 3 (IGFBP3), matrix metalloproteinases (MMPs) 2 and 9, plasminogen activator urokinase (PLAU), and the KIT receptor tyrosine kinase. Further analysis revealed that hispolon down-regulated the protein levels of EGFR, PLAU, and KIT [270].

Several studies have shown that hispolon affects estrogen signaling. Indeed, hispolon suppresses the protein level and transcriptional activity of Erα [271]. Another study showed that hispolon inhibits ERα/β, aromatase, and cyclooxygenase-2 (COX-2) in breast cancer models [272]. However, Wang et al. demonstrated the direct interaction of hispolon with ERα and ERβ, showing a biphasic mode of hispolon-mediated estrogenic activity. At low concentrations, hispolon stimulates ERs; at high concentrations, it exhibits antagonistic activity [273]. This bi-phasic mode of action was also demonstrated in [272]. Taken together, these results suggest that it may be dangerous to use hispolon to treat estrogen receptor-positive breast cancer.

Despite multiple studies and pharmacological activities, the direct targets of hispolon beyond ERα/β remain elusive.

The main pharmacokinetic challenge of hispolon is its poor water solubility, which results in low bioavailability and limits its clinical use. A key finding is that forming an inclusion complex with sulfobutylether-β-cyclodextrin (SBEβCD) improves hispolon’s solubility 15-fold, increasing it from 2.11 mM to 29.6 mM [274]. Furthermore, encapsulating this complex within sterically stabilized liposomes (HSC-SL) creates a stable delivery system with high encapsulation efficiency (>90%) and provides a sustained release profile [274]. A formulation of hispolon within liquid crystalline nanoparticles (HP-LCNPs) was developed and demonstrated excellent stability and a biphasic drug release profile. This formulation resulted in a 4.8-fold increase in oral bioavailability and significantly improved therapeutic outcomes in an animal model of liver cancer [275].

***Other types of low-molecular weight compounds derived from mushrooms***.

***Cordycepin***, (3′-deoxyadenosine) is the primary component of *Cordyceps militaris*, a well-known traditional Chinese medicinal fungus (Figure 8). It exhibits anti-neoplastic, anti-inflammatory, and cardioprotective and neuroprotective activities [276]. Various studies have examined the anti-tumor properties of cordycepin and its effects on the cell cycle, apoptosis, migration, metabolic rewiring, and signaling pathways (reviewed in [277]).

Pan et al. have demonstrated that cordycepin induces apoptosis in Leydig tumor cells (testicular cancer) through p38 MAPK. In addition, it is much less toxic to primary Leydig cells (non-cancerous) [278]. Importantly, cordycepin significantly reduced xenograft growth at a dose of 20 mg/kg. However, in cells resistant to cordycepin, it activated AKT/mTOR, ERK, and JNK kinases. Additionally, the authors proposed that cordycepin induces unfolded protein response-dependent cell death, though this occurs only at high concentrations [279].

Several studies have demonstrated that cordycepin, an adenosine analog, is an agonist of adenosine receptors (ADORA). Of the four ADORAs, cordycepin has been shown to be a ligand for ADORA2A [280,281] and ADORA3 [282,283,284] receptors that mediate cordycepin-mediated anticancer effects in several cancer models.

However, the contradictory role of cordycepin-mediated activation of ADORAs in its multiple antitumor properties is unclear. Typically, activation of the ADORA2A and ADORA2B receptors, as well as treatment of cancer cells with adenosine, is associated with various oncogenic properties, including tumor growth, metastasis, and immunosuppression. Furthermore, ADORA2A and ADORA2B receptor expression is often linked to a poor prognosis for cancer patients [285,286]. In contrast, ADORA3 agonists suppress the growth of various cancer cells and animal models while increasing NK cell activity [287]. Thus, more studies are needed to elucidate the molecular mechanisms of cordycepin-mediated antitumor effects and the role of ADORA receptors in this phenomenon.

In both the hepatocellular carcinoma cells and the xenograft model, cordycepin suppressed glycolysis, which was associated with the downregulation of three important glycolytic enzymes: HK2, PKM2, and LDHA [288]. Interestingly, the authors also showed that cordycepin simultaneously activates AKT and AMPK. This seems contradictory, but it has also been reported [278].

In contrast, cordycepin was shown to elicit ROS-dependent inactivation of the PI3K/Akt pathway in bladder cancer cells, inducing both intrinsic and extrinsic apoptotic pathways [289]. Additionally, in glioma and osteosarcoma cell models, cordycepin suppressed AKT and induced AMPK, thus sensitizing malignant cells to temozolomide [290] and cisplatin [291], respectively. In lung carcinoma, cordycepin significantly ameliorated AKT/mTOR signaling while induced AMPK activation [292]. Finally, cordycepin-mediated AKT inhibition was demonstrated in gastric cancer [293], and cholangiocarcinoma [294].

Another negative effect of cordycepin on metabolic reprogramming in tumors was demonstrated in cholangiocarcinoma, both *in vitro* and *in vivo*. Cordycepin displayed hypolipidemic effects, suppressing SREBP1—the key transcriptional regulator of fatty acid biosynthesis—through the inhibition of the AKT/mTOR signaling pathway [294]. This led to the downregulation of enzymes involved in fatty acid biosynthesis (FASN and ACC1) and suppressed migration and metastasis.

Thus, the aforementioned studies have demonstrated that cordycepin mediates AMPK activation and AKT/mTOR inhibition. This phenomenon can apparently be explained by the fact that AMPK is a direct target of cordycepin.

First, it was shown that cordycepin physically interacts with the γ subunit of AMPK [295]. subsequently, it was demonstrated that, in ovarian cancer cells, cordycepin is transported into the cells via the equilibrative nucleoside transporter 1 (ENT1). The authors revealed that cordycepin induces autophagic cell death via ENT1-mediated transport rather than through the activation of ADORA2A and ADORA2B. Once inside the cells, cordycepin is converted into a monophosphorylated form that activates AMPK. Finally, at a dose of 25 mg/kg, cordycepin significantly reduced the volume of ovarian cancer xenografts [296]. In lung carcinoma models, cordycepin preferentially suppressed cells with mutated EGFR. It was as effective as afatinib and more effective than gefitinib, dramatically reducing xenograft growth [292]. The authors linked this to strong AMPK activation.

Recently, Hawley and his co-authors proposed a model for cordycepin-mediated activation of AMPK. They suggested that a 100 μM dose of cordycepin induces AMPK activation, which correlates with cellular cordycepin monophosphate content. This monophosphate mimics all of AMP’s effects on AMPK [297].

In breast cancer models, cordycepin decreased cell growth by suppressing the Hedgehog signaling pathway and inhibiting GLI transcriptional activity [298]. This was associated with the upregulation of E-cadherin and the downregulation of EMT markers N-cadherin, SNAIL, SLUG, and ZEB1. In another study, cordycepin dramatically reduced the growth of xenografts of triple-negative breast cancer cells [299]. Transcriptomic analysis of these xenografts showed that genes involved in Hedgehog signaling pathways were the main group of genes down-regulated by cordycepin. Further studies revealed the strong cordycepin-mediated suppression of hedgehog signaling pathway components (SHH, SMO, Ptch1, and Gli1 and Gli2) and EMT markers (N-cadherin, Snail, Slug, and Zeb1) [299].

Finally, several studies have demonstrated the positive effects of cordycepin on the upregulation of anti-tumor immunity [300,301,302].

The key quantitative pharmacokinetic challenge for cordycepin is its extremely short plasma half-life of less than 1.5 min in mice due to rapid enzymatic deamination [303,304]. When administered orally to rats, intact cordycepin was not detected in the plasma. Instead, its deaminated metabolite, 3′-deoxyinosine (3′-dI), was absorbed. It reached a maximum concentration Cmax of 7.36 μM with a time to maximum concentration T_max_ of 30 min and a terminal half-life of approximately 1.49 h [305].

Current research focuses intensely on ADA inhibition, structural analogs, and nano-delivery systems to overcome this barrier alongside a novel understanding of its metabolic activation pathway [304].

***Illudins***. The illudins, predominantly illudin S and illudin M, are a family of fungal sesquiterpenes initially isolated from the *Omphalotus olearius* mushroom. A comprehensive investigation into their potential antitumor properties has been conducted, encompassing a range of tumor cell types. However, these substances were less effective in animal models, and their potential as anticancer medications was limited by their low selectivity for cancerous cells compared to healthy cells. This was accompanied by substantial systemic toxicity [306].

Semi-synthetic derivatives called “acylfulvenes” were developed to expand the therapeutic window of this class of natural products. These derivatives have been the focus of numerous clinical studies in the context of tumors that are resistant to treatment and multiple pharmaceutical interventions. Irofulven, a highly promising acylfulvene analog, advanced to Phase III clinical trials for treating various cancers. However, it was subsequently discontinued due to ineffectiveness [307].

It is widely accepted that illudins primarily induce cell cycle arrest and apoptosis by alkylating and forming DNA adducts. For example, their research focuses on the transcription-coupled nucleotide excision repair (NER) process and how it sensitizes chronic lymphocytic leukemia cells to fludarabine [308,309].

We found no specific data on the bioavailability, time to maximum concentration (T_max_), or half-life (T_1/2_) of Illudin S. However, in a phase I trial, the mean plasma half-life of its derivative, Isofulven, was 4.91 min after a 30 min intravenous (IV) infusion, with rapid clearance [310].

***Hispidin***, 6-(3,4-dihydroxystyryl)-4-hydroxy-2-pyrone (Figure 8), is a polyketide constituent from *Inonotus hispidus* and mushrooms of genus *Phellinus* (*P. linteus* also known as “sanghuang”, *P. ignarius*, *P. baumii*, *P. harmala*, and *P. sensulato*). It has been shown to have anticancer, antibiotic, cholesterol-lowering, anti-diabetic, and neuroprotective effects [311].

Although most studies focus on the pharmacological activities of hispidin in non-cancer cells, several studies have addressed its anti-neoplastic properties.

It has been shown that hispidin is more cytotoxic toward cancer cells than toward normal cells and inhibits protein kinase C β (PKCβ, IC_50_ = 2 μM), which plays a crucial role in carcinogenesis [312]. In colon cancer cells, hispidin induces apoptosis dependent on elevated ROS production [313].

In prostate cancer models, hispidin induced cell cycle arrest and apoptosis. It also suppressed migration and invasion and decreased the protein levels of the androgen receptor (AR) and metalloproteinases 2 and 9 (MMP2 and MMP9) [314]. Other research on prostate cancer has demonstrated that hispidin induces apoptosis and ferroptosis and inhibits AKT signaling while upregulating MAPK (p38, ERK, and JNK) and NF-κB [315].

In pancreatic adenocarcinoma cell models, hispidin increased ROS production, reduced stemness, and sensitized the cells to gemcitabine. Mechanistically, hispidin significantly suppressed the protein levels of the common cancer stem cell markers CD44, Nanog, and SOX2 [316].

There is no available data on hispidin’s pharmacokinetics in humans or animals, its oral bioavailability, or its T_max_ and T_1/2_.

As the body of literature on mushroom-derived compounds continues to grow, the *in vivo* evidence has become more diverse, covering various tumor models, administration methods, and endpoints. This variability makes direct comparison across studies challenging. However, such comparison is essential for recognizing consistent pharmacological patterns and identifying the most promising compounds. Table 4 consolidates representative in vivo findings from recent studies and emphasizes key experimental parameters, such as animal models, biological outcomes, and mechanistic markers. Aligning these data side by side provides a clearer framework for evaluating the comparative potency, safety, and mechanistic convergence of mushroom-derived metabolites in different research contexts.

## 7. Clinical Experience of Using Mushrooms in Oncology

Numerous clinical studies have examined the use of medicinal mushrooms as an adjunct to standard anticancer therapy. These studies have primarily focused on immune modulation, symptom burden, and quality-of-life outcomes. However, the quality of the studies, the size of the samples, and the clinical endpoints vary widely. Recent reviews provide comprehensive overviews [14,322]. Here, we summarize the key medicinal mushroom species that have been investigated in clinical trials. We emphasize the study design, primary endpoints, and methodological limitations.

Across studies, medicinal mushroom preparations have most consistently been associated with changes in immune parameters, symptom burden, and quality-of-life scores. However, evidence for effects on survival remains limited, inconsistent, and highly dependent on the context [14]. While some trials report reduced chemotherapy toxicity or favorable cytokine profiles, relatively few randomized controlled trials demonstrate clear disease-modifying effects. These effects are largely confined to specific populations and treatment settings [322].

In some countries, certain medicinal mushroom-derived products have been approved for clinical use as adjunctive therapies and incorporated into routine practice in specific settings. For instance, polysaccharide-K (PSK, Krestin) has been approved and is widely used in Japan as an oral adjuvant immunochemotherapy for gastric and colorectal cancer, particularly in the postoperative setting [323]. *Lentinula edodes* (shiitake) is commonly used in China within integrative oncology frameworks, often alongside conventional anticancer treatments.

Zhang et al. reviewed 135 clinical studies encompassing 9474 patients treated with lentinan for various malignancies between 2004 and 2016 [324]. Most of the studies examined the use of lentinan alongside chemotherapy and found higher objective response rates, improved immune indices, and better quality-of-life measures for lung, gastric, and hepatic cancers compared to chemotherapy alone. Several trials in lung, colorectal, and gastric cancers suggested an association with improved clinical outcomes, which was largely attributed to enhanced immune function and reduced treatment-related toxicity [325]. However, most of these studies were small and single-center, with heterogeneous designs, comparators, and endpoint selection. There was also limited use of randomized controls and survival as a primary endpoint. Consequently, while these data support a potential supportive and immunomodulatory role for lentinan in certain situations, definitive conclusions regarding disease-modifying efficacy or survival benefits are limited.

Multiple clinical investigations suggest that lentinan can modulate immune function in cancer patients. Some reports describe cytokine changes indicative of enhanced Th1-type activity following chemotherapy. However, direct assessment of the Th1/Th2 balance in clinical settings remains limited [9]. In a retrospective cohort study of 73 patients with non-small cell lung cancer (NSCLC) who received vinorelbine–cisplatin chemotherapy, patients who received adjunctive lentinan had higher proportions of CD3^+^CD8^+^ T cells and CD3^+^CD56^+^ NKT cells, lower proportions of CD4^+^CD25^+^ regulatory T cells, and higher plasma levels of IFN-γ, TNF-α, and IL-12 than patients who received chemotherapy alone. These immunological changes are consistent with enhanced cellular immune activity and a more proinflammatory cytokine profile. This has been interpreted as a shift toward Th1-type immunity in the lentinan-treated group [324].

Another *Lentinula*-derived polysaccharide fraction, AHCC, has also been evaluated as an adjuvant in small clinical trials [326,327]. For instance, a prospective, nonrandomized, controlled trial of 75 patients with unresectable pancreatic ductal adenocarcinoma (PDAC) in Japan compared gemcitabine alone (*N* = 40) with gemcitabine plus AHCC (*N* = 35). The trial reported an attenuated increase in C-reactive protein (CRP) and a decline in albumin, as well as some treatment-related adverse effects (notably, taste distortion), with potential benefits to quality of life. However, the short two-month intervention, surrogate endpoints, and lack of randomization and blinding limit the strength of these findings [328].

A recent systematic review [322] identified 39 clinical studies of medicinal mushroom supplements in cancer, each with at least 10 patients, published between 2010 and 2020. Of these studies, 15 were randomized controlled trials (RCTs), 13 were retrospective, and 11 were prospective non-randomized trials. Many of the studies reported positive immunological or quality-of-life outcomes and reduced symptom burden; however, only seven demonstrated a survival benefit. Two hepatocellular carcinoma (HCC) trials and one breast cancer study evaluated Huaier granules (*Trametes robiniophila* Murr.), and four gastric cancer RCTs investigated PSK as an adjuvant. PSK was the most commonly studied constituent among eleven mushroom-derived preparations [322]. Overall, the conclusions that can be drawn are constrained by the heterogeneity in design and endpoints, as well as the limited blinding and randomization in many studies.

*Trametes versicolor* (Yun Zhi/Kawaratake, or “turkey tail”) has been used in East Asia for centuries. Its primary clinical preparations are the polysaccharopeptides PSK (Krestin) and PSP. These preparations are produced by different strains and have different polysaccharide-to-peptide compositions (approximately 60:40 for PSK and 90:10 for PSP) [329]. Venturella et al. reported that at least 12 *T. versicolor*–based drugs have been approved by the Chinese State Administration of Food and Drugs (SAFD) [9].

The strongest clinical evidence for PSK is in the adjuvant setting, but it remains mixed and context-dependent. Several observational gastric cancer studies evaluating the addition of PSK to postoperative chemotherapy reported associations with improved recurrence-related outcomes in selected subgroups. For instance, in a clinical cohort study of 349 patients with stage II/III gastric cancer who underwent curative resection, the addition of PSK to postoperative chemotherapy was examined in terms of recurrence-free survival (RFS). In the prespecified subgroup of patients with MHC class I–negative tumors and/or pN2+ nodal status, PSK was associated with higher three-year RFS compared to chemotherapy alone [323]. Similarly, other retrospective analyses (including larger databases) reported PSK as an independent prognostic factor for overall survival in multivariable models. These models suggest effect modification by tumor or immune context (e.g., PD-L1 status) and accompanying shifts in NK/NKT cell proportions in small immunophenotyping subsets [330,331]. However, the observational design of these studies, the heterogeneity of chemotherapy regimens, and the frequent reliance on subgroup analyses limit causal inference and generalizability.

Although evidence from randomized trials is more informative, it is still limited. In a randomized adjuvant trial of stage II–III colorectal cancer patients (*N* ≈ 205), adding PSK to tegafur/uracil-based chemotherapy was associated with improved five-year disease-free and overall survival compared with chemotherapy alone. Nevertheless, the use of older oral fluoropyrimidine protocols may reduce the applicability of these results to contemporary intravenous regimens [332]. A systematic review and meta-analysis of 13 double-blind, randomized trials comparing conventional anticancer therapy with and without PSK reported an approximately 9% absolute reduction in five-year mortality in PSK treatment groups, primarily in cohorts with breast, gastric, and colorectal cancers. However, substantial heterogeneity in populations and treatment regimens warrants cautious interpretation [333].

Beyond survival endpoints, several small randomized studies have evaluated immunological surrogates. For example, a randomized controlled trial (RCT) of 30 patients with cT3/T4 rectal adenocarcinoma examined the effects of preoperative chemoradiotherapy (CRT) with or without concurrent PSK. Compared with CRT alone, the PSK group showed higher increases in circulating natural killer (NK) and cytotoxic T-lymphocyte counts in peritumoral and normal mucosa. There was also a reduction in serum immunosuppressive acidic protein levels [334]. Another small RCT in Japan involved 21 patients with resected stage III gastric cancer. Adjuvant PSK combined with tegafur/uracil (UFT) was associated with a higher three-year overall survival rate than UFT alone (62.2% vs. 12.5%; *p* = 0.038). PSK was also associated with a postoperative reduction in the proportion of CD57^+^ T cells, a poor prognostic marker. However, other lymphocyte subsets and the Th1/Th2 balance did not differ pre-treatment [335]. Due to the small sample sizes, surrogate endpoints, and variability in study design, these findings should be viewed as supportive immunomodulatory signals rather than as definitive evidence of a disease-modifying benefit.

Huaier (*Trametes robiniophila* Murr.; “Huai Qihuang”) has been used in traditional Chinese medicine for approximately 1600 years. Clinical studies have primarily examined the use of oral Huaier granules as an adjunct to standard cancer treatments for various tumor types, employing diverse study designs and endpoints [336]. In breast cancer, a single-center retrospective cohort study compared conventional therapy plus Huaier granules (*N* = 140) with conventional therapy alone (*N* = 144) and reported longer disease-free survival in the Huaier group. There were also improvements in the Karnofsky Performance Scale, reductions in circulating tumor markers, and improvements in patient-reported emotional symptom burden. However, the non-randomized design and potential residual confounding factors limit causal inference regarding long-term benefits [337].

In gastric cancer, a single-center retrospective analysis of stage IIb disease (*N* = 126) compared postoperative tegafur, gimeracil, and oteracil potassium (TGOP) chemotherapy with (*N* = 54) and without (*N* = 72) Huaier granules. The analysis reported modestly longer disease-free and overall survival in the Huaier-treated group. However, the observational design and incomplete control for confounders mean the findings should be considered hypothesis-generating rather than confirmatory [338].

In a highly specific post-transplant setting, Zhou et al. retrospectively evaluated 36 hepatocellular carcinoma (HCC) patients undergoing liver transplantation and compared two regimens: a sirolimus-based regimen combined with thymalfasin plus Huaier versus a tacrolimus-based regimen without Huaier. The combination regimen was associated with longer recurrence-free intervals and overall survival, as well as changes in immune parameters. However, the small sample size and multi-component intervention preclude attributing clinical effects specifically to Huaier, limiting the generalizability of the findings beyond the context of post-transplant immunosuppression [339].

The interventional evidence includes an RCT of unresectable primary HCC (*N* = 62), which compared TACE with gelatin sponge particles plus lobaplatin with and without Huaier granules. The Huaier group had higher 12-month overall survival and objective tumor response rates, though median overall survival did not differ significantly. The small sample size and limited follow-up time prevent definitive conclusions about sustained survival benefits [340].

The strongest evidence to date is a multicenter, phase IV, randomized controlled trial (RCT) of 1044 patients with HCC after curative resection that compared two years of adjuvant Huaier granules versus no adjuvant therapy. Huaier granules significantly improved recurrence-free survival and reduced extrahepatic recurrences. However, interpretation of the effects on overall survival should be cautious, given the endpoint hierarchy and follow-up considerations [341].

Longer-term, real-world evidence from a large, retrospective cohort study (*N* = 1111) with propensity score matching reported an association between Huaier use after curative resection and higher five-year overall and recurrence-free survival. However, despite matching, the observational design leaves room for residual confounding, and the results should be interpreted as supportive, but not definitive [342].

Finally, a recent systematic meta-analysis of 29 RCTs (*N* = 2206) across several cancers assessed immunological readouts and found that adding Huaier to conventional therapy was associated with increases in CD3^+^, CD4^+^, NK-cell percentage, and the CD4^+^/CD8^+^ ratio, while changes in CD8^+^ T cells were not significant; substantial heterogeneity and variable trial quality limit firm conclusions regarding the clinical relevance of these immune shifts [343].

Overall, the clinical data suggest that Huaier granules may improve recurrence-related outcomes in certain situations, particularly after resection of hepatocellular carcinoma (HCC), and may modulate immune markers. However, the strength of the evidence varies by indication and study design. Additional rigorously designed trials with pre-specified clinical endpoints are needed before broad clinical recommendations can be made.

*G. lucidum* (Lingzhi/Reishi) is widely used as a medicinal mushroom, and its polysaccharide-rich preparations (GLPs) are primarily investigated for their immunomodulatory and supportive effects rather than for their direct tumor cytoreductive effects [344]. In a randomized, double-blind, placebo-controlled, multicenter trial of 68 patients with advanced lung cancer, oral Ganopoly^®^ was associated with improvements in performance status (KPS) and select immune parameters. However, antitumor efficacy was mainly reported as disease stabilization or response metrics, without definitive evidence of tumor regression. The short duration of the trial and reliance on supportive endpoints limit the ability to infer disease-modifying benefits [345]. In a separate, single-arm, prospective study of 34 patients with advanced-stage cancer, Ganopoly administration was associated with increased NK activity and other immune parameters versus baseline. However, the absence of a control group precludes causal attribution [346]. A small, non-randomized, prospective study of 40 breast cancer patients receiving chemotherapy reported changes in inflammatory and immune cytokines with the addition of *G. lucidum* supplementation, but it lacked randomization, blinding, and clinical efficacy endpoints [347]. Consistent with these limitations, a 2016 Cochrane systematic review identified five randomized controlled trials (*N* = 373) and concluded that there is limited evidence suggesting possible improvements in treatment response and quality of life when *G. lucidum* is used adjunctively. However, there are methodological concerns, and no long-term survival data were recorded across the included trials [348].

The sun mushroom, *Agaricus blazei* Murill, is native to Brazil but popular in Japan. According to Takaku et al., Japan produces 100,000–300,000 kg of dried *A. blazei* every year. Between 300,000 and 500,000 people have used *A. blazei* for cancer prevention or alongside chemotherapy after tumor removal as part of a complementary alternative medicine program [349]. The main biologically active constituents of *A. blazei* are β-(1 → 3)-D-glucan, β-(1 → 4)-D-glucan, β-(1 → 6)-D-glucans, and several glycoproteins [350].

In a randomized, placebo-controlled, blinded trial of 100 gynecological cancer patients receiving platinum-based chemotherapy, those who received *A. blazei* Murill Kyowa (ABMK) (*N* = 39) had significantly higher natural killer (NK) cell activity than those who received a placebo (*N* = 61). However, no significant differences were observed in other immune cell subsets. However, the trial did not assess tumor response or survival outcomes, nor were quality-of-life measures evaluated using validated instruments. Thus, conclusions regarding disease-modifying efficacy are limited [351].

In a randomized, double-blind, placebo-controlled trial involving 40 patients with multiple myeloma undergoing high-dose chemotherapy followed by autologous stem cell transplantation, adjunctive treatment with AndoSan^®^ for two months was associated with significant changes in immune parameters. These changes included increased plasma levels of IL-1ra, IL-5, and IL-7, as well as higher proportions of regulatory T cells and plasmacytoid dendritic cells compared with the placebo group. However, the study was not powered to assess clinical endpoints such as relapse, progression-free survival, or overall survival. The small sample size and short follow-up period also limit conclusions regarding disease-modifying efficacy [352].

A retrospective cohort study of 217 patients with pancreatic ductal adenocarcinoma undergoing curative pancreatectomy by Lee et al. examined the medicinal mushroom *Phellinus linteus* [353]. The study showed that *P. linteus* medication was the only independent predictor of completion of planned adjuvant chemotherapy (*p* = 0.039) after propensity score adjustment. Completion of adjuvant therapy was associated with significantly longer disease-free and overall survival in the *P. linteus* group than in the group not receiving *P. linteus. P. linteus* use was also linked to fewer chemotherapy dose reductions or early cessation. However, the retrospective design, single-institution setting, and lack of randomization limit causal inference about the direct antitumor effect of *P. linteus* [353].

Importantly, not all randomized evidence supports the clinical benefits of medicinal mushroom–derived adjuvants. Okuno et al. conducted a prematurely closed phase III randomized trial in stage II rectal cancer patients (*N* = 111), comparing surgery alone to adjuvant UFT plus PSK. Disease-free survival was lower in the UFT+PSK group, though overall survival was similar between groups. However, early trial termination and limited statistical power preclude definitive conclusions regarding efficacy or harm [354].

Overall, clinical evidence suggests that certain medicinal mushroom-derived preparations, particularly PSK, lentinan, Huaier, and *Ganoderma* polysaccharides, may provide supportive benefits in oncology, primarily through immune modulation, improved treatment tolerance, and enhanced quality of life. However, evidence for direct disease-modifying effects or survival benefits is inconsistent, context-dependent, and largely confined to specific preparations and clinical settings. Most positive findings come from observational studies, subgroup analyses, or trials using surrogate immunological endpoints. This underscores the need for well-controlled, adequately powered randomized studies with prespecified clinical endpoints to determine these agents’ true therapeutic role in modern oncology.

## 8. Safety and Limitations of Using Medicinal Mushrooms in Clinic

In this final chapter, we consider the main obstacles limiting the use of medical mushrooms in clinical oncology. First of all, the main limitation is related to insufficient clinical data on the efficiency and safety of using mushrooms as adjuvants. While *in vitro* and *in vivo* models have demonstrated the potential of mushrooms as anticancer agents, most of the evidence comes from preclinical studies. Clinical evidence in cancer patients remains limited.

Numerous clinical studies have revealed an absence or minimal occurrence of adverse effects (AEs) from the clinical application of reishi, shiitake, turkey tail, huaier, and *Cordyceps sinensis* [26,355,356,357,358,359,360]. However, more specific studies on the safety of these mushroom-derived substances should be conducted on patients with certain types of cancer.

Nevertheless, a number of clinical trials and case reports have documented AEs. This necessitates careful interpretation because specific AEs may be attributable to the underlying condition or concomitant treatment [14]. Utilizing placebo-controlled groups and double-blind assessments is imperative for accurately interpreting adverse events.

For example, Ohno et al.’s phase I study of patients with cancer in remission who took an *Agaricus blazei* Murill supplement reported AEs in 9 of 78 patients (12%). Most of them were associated with the digestive tract and manifested as symptoms such as nausea and diarrhea. Notably, one patient experienced food allergies associated with liver dysfunction [361]. Hishamochi et al. documented two cases of liver injury associated with *Agaricus blazei* Murill. This mushroom has been found to resemble autoimmune hepatitis [362].

Administering elevated doses of *G. lucidum* spore extract has been shown to increase cancer antigen (CA) 72-4 levels. However, no harmful effects on bodily organs have been observed [363]. Oxalate nephropathy, a condition characterized by the presence of oxalate crystals in the kidneys, was observed in a 72-year-old Japanese woman diagnosed with liver cancer. This occurrence was attributed to her consumption of chaga mushrooms [364].

Another clinically significant safety concern is the potential for herb-drug interactions between mushroom-derived products and approved medications, which could have beneficial or harmful effects in an unpredictable manner. Preclinical studies have shown that several mushroom species and their purified constituents modulate cytochrome P450 enzymes (notably CYP1A2, CYP2E1, and CYP3A4) and drug transporters. For instance, *Ganoderma lucidum* polysaccharides have been reported to inhibit multiple CYP450 isoforms *in vitro* and alter the pharmacokinetics of probe drugs in animal studies. This suggests a potential for clinically relevant drug–drug interactions [365,366]. In addition, *T. versicolor*-derived polysaccharide peptide (PSP) competitively inhibited the CYP1A2- and CYP3A4-mediated metabolism of model probe substrates in human liver microsomes *in vitro* [367].

Agaricus-based preparations have been shown to inhibit P-proteoglycan in Caco-2 epithelial cells *in vitro* [368]. These effects raise the possibility of altered drug exposure for agents with narrow therapeutic windows, including cytotoxic chemotherapies, tyrosine kinase inhibitors, anticoagulants, and immunosuppressants. This could lead to a loss of efficacy or increased toxicity.

Pharmacodynamic interactions should also be considered. For example, *Ganoderma lucidum* has been reported to exhibit antiplatelet and anticoagulant activity *in vitro* and *in vivo* [369], which could increase the risk of bleeding when combined with warfarin or antiplatelet drugs. Patients receiving simvastatin, montelukast, or anticoagulant therapy are advised to discontinue *Ganoderma lucidum* products at least seven days before surgery [370,371]. As previously discussed, mushroom-derived immunomodulatory substances, such as PSK, PSP, lentinan, and AHCC, have been shown to enhance Th1-type responses and natural killer (NK) cell activity in preclinical models [372,373,374]. These effects may create the potential for synergy with immunotherapy, but they also raise the theoretical risk of exacerbating immune-related adverse events or counteracting immunosuppressive medications. Although formal clinical pharmacokinetic studies are limited and often underpowered to detect subtle drug–herb interactions, the underlying biological plausibility and isolated clinical reports warrant cautious interpretation of safety outcomes.

On the other hand, administering mushrooms has been observed to reduce AEs associated with chemotherapy, decrease hematological toxicity, and mitigate hepatotoxicity. For example, ingesting AHCC (active hexose correlated compound, a commercially available polysaccharide fraction derived from shiitake) has been shown to reduce the incidence of adverse effects caused by gemcitabine. Additionally, AHCC has been shown to prevent taste impairments, which can reduce the prevalence of anemia and anorexia during chemotherapy in patients with unresectable pancreatic ductal adenocarcinoma [328]. The administration of AHCC in conjunction with chemotherapy for breast cancer has been demonstrated to result in a reduction in neutropenia-related events, thereby decreasing the necessity for granulocyte colony-stimulating factor usage [375]. The combination of lentinan and chemotherapy has been shown to reduce the incidence of AEs in patients with ovarian and gastric cancer [376,377]. In another study, patients with breast cancer who received *Agaricus sylvaticus* exhibited improved nutritional status and reduced gastrointestinal symptoms, such as nausea, vomiting, and loss of appetite, compared to those who received a placebo [378]. Several studies have documented an enhancement of quality of life and emotional well-being with the use of reishi supplements [360,379].

Despite promising outcomes, a number of factors have the potential to restrict the validity of the clinical research that has been conducted thus far. Salient features of these studies include the paucity of sample size, the absence of a control or placebo group, numerical disparity in group sizes, limited replication, poor reporting of adverse events, inadequate statistical methods, and poorly described results [9].

Additionally, AEs related to mushroom products have been reported, including drug or herb/supplement interactions with mushrooms, organ toxicity, metabolic disorders, and tumor growth. For example, maitake (*Grifola frondosa*) extract has been shown to increase the INR blood clot test score and increase the risk of bleeding when taken with warfarin [380]. Although low-grade AEs were observed with few major AEs, caution should be exercised when interpreting the results due to the retrospective nature of some studies. While numerous studies demonstrate the efficacy of mushroom products in promoting favorable immune outcomes, contradictory findings regarding their immunosuppressive properties and inconsistent immune responses have also emerged [352,381]. These conflicting findings present challenges in providing clinical guidance.

Moreover, the lack of regulation regarding supplements may raise concerns about toxicity due to the potential for contamination and inadequate quality control of product ingredients. Patients may be exposed to excessive amounts of harmful compounds, such as heavy metals or microbes, in supplements. Despite mounting interest and supporting evidence, medical education offers limited training on herbs and supplements. This presents challenges for oncologists who are trying to communicate reliable information to their patients. The current body of evidence is insufficient to support the routine use of mushrooms in cancer patients. Consequently, the intricate interplay between mushrooms and the immune system in cancer requires further exploration. To substantiate the hypothesis that combining mushrooms with standard cancer therapy is efficacious and safe, large-scale, randomized clinical trials are required.

Finally, a fundamental safety concern regarding wild-grown mushrooms is the risk of misidentifying them. Several highly toxic species of mushrooms bear a superficial resemblance to edible or medicinal varieties. For instance, the deadly *Galerina marginata* can be mistaken for medicinal *Psilocybe* species or other small brown mushrooms. Conversely, the toxic *Omphalotus olearius* (jack-o’-lantern mushroom) is sometimes confused with edible chanterelles (*Cantharellus* spp.). In the worst-case scenario, consuming misidentified wild mushrooms can lead to severe hepatorenal toxicity and death—a risk that outweighs any unverified anticancer benefits. This underscores the critical importance of obtaining mushroom products from reputable, cultivated sources with verified identity.

## 9. Conclusions and Perspectives

Extensive research has documented the antineoplastic properties of mushrooms, which are derived from two primary sources: high-molecular-weight cell wall components (polysaccharides and proteoglycans) and low-molecular-weight secondary metabolites (terpenes, phenolic compounds, etc.). The primary anticancer mechanism is indirect and immunological, driven by polysaccharides and proteoglycans. These compounds act as immunomodulators, binding to pattern-recognition receptors such as Dectin-1 and TLR2/4 on intestinal immune cells. This triggers a systemic cascade that enhances both innate and adaptive immunity. It promotes dendritic cell maturation, regulates macrophage and T-helper cell polarization, increases natural killer cell populations, and elevates key cytokines, such as IFN-γ, TNF-α, and IL-12. Although the immune boost is well-established, the potential involvement of complement receptor 3 (CR3) in mushroom-mediated cytotoxicity, a critical pathway in therapy-induced cell killing, remains an open and clinically significant question.

There is evidence of the direct anticancer activity of mushroom polysaccharides and proteoglycans. However, the physiological significance of this phenomenon is questioned due to the lack of physical contact between these substances and tumor cells throughout the body, except in the case of colorectal cancer. We hypothesize that, in this case, local contact enables triple action: enhanced systemic immunity, ameliorated intestinal inflammation, and direct tumor suppression. Additionally, the molecular mechanisms of the direct anticancer activities of polysaccharides and proteoglycans should be elucidated.

In contrast to high-molecular-weight cell wall components, the direct anticancer activities of mushrooms are mainly conferred by a set of low-molecular-weight compounds (secondary metabolites). These compounds inhibit key signaling pathways and affect different hallmarks of malignant cells. The potential synergy between high-molecular-weight cell wall components (polysaccharides and proteoglycans) and low-molecular-weight secondary metabolites, combining immunomodulation with direct cytotoxicity, is a key area for preclinical investigation, particularly with “double” extracts, combining both of them. In addition, specific fungal immunomodulatory proteins (FIPs, such as LZ-8 and GMI) directly affect cancer cell signaling and represent promising adjuvants.

The vast majority of clinical trials have used purified polysaccharides and proteoglycans derived from mushrooms, suggesting that boosting the immune system is the main cause of the anticancer activity of mushrooms. From a clinical perspective, the evidence of a survival benefit is inconsistent, though it is strongest for specific, purified interventions, such as PSK (*Trametes versicolor*) for gastric/colorectal cancer and Huaier granules (*Trametes robiniophila*) for hepatocellular carcinoma. Many other species require large-scale, confirmatory randomized controlled trials. In addition to these immune effects, a set of secondary metabolites exerts direct anticancer activity by inhibiting crucial signaling pathways within malignant cells. Large, well-designed, randomized controlled trials with standardized clinical endpoints are highly anticipated to determine the therapeutic potential of medicinal mushrooms in oncology.

## Figures and Tables

**Table 1:** Results of some in vivo studies on mushroom polysaccharides and proteoglycans.

Mushroom (Source) and Substance	Primary Immune Receptor(s)	Key Immune Cell Targets	Major Immunological Outcome(s)	Alterations in Cytokines/Cell Markers	Reference
*Lentinula edodes* (Shiitake)—Lentinan (β-glucan) 100 μg/mouse/day	Dectin-1, TLR4	Intestinal epithelial cells, Macrophages, DCs, T cells	Th1 polarization, suppression of intestinal inflammation, enhanced antigen presentation.	↑ IL-12, ↑ IFN-γ, ↓ TNF-α/IL-6 (in colitis), CD4^+^ Th1 differentiation.	[80,81]
*Armillaria mellea* β-(1→6)-glucans 10 mg/kg/day and 20 mg/kg/day, i.p., for a total of 10 days.	TLR2	Macrophages (M2)	Shift from M2-like to M1-like macrophages.	Activation of Akt/NF-κB and MAPK pathways	[86]
*Trametes versicolor* (Turkey Tail)—PSK (Proteoglycan) 2 mg/mouse 3 times per week for 4 weeks	TLR4, TLR2	Macrophages, DCs, NK cells, B cells, T cells	Macrophage activation, NK cell activation, B cell proliferation, support of CD8+ T cell activity.	↑ TNF-α, ↑ IL-6, ↑ IgM/IgG1, ↑ NK cell activity.	[85]
*Ganoderma lucid-um* (Reishi) crude extract (3 and 6 mg/kg once daily for 2 weeks)		Splenocytes, Macrophages, NK cells	Enhanced splenocyte proliferation, increased macrophage phagocytosis, and elevated NK cell activity.	↑ IL-6, ↑ IFN-γ	[94]
*Agaricus blazei*—Polysaccharide fractions 2 mg/mouse 3 times per week for 4 weeks	TLR2, Dectin-1	Macrophages, MDSCs	Repolarization of MDSCs/M2 macrophages to M1-like phenotype.	↑ IL-6, ↑ IL-12, ↑ TNF-α, ↑ iNOS, ↑ CD86, ↑ MHC II, ↑ pSTAT1.	[87]
*Grifola frondosa* (Maitake)—D-fraction/Polysaccharides 20 mg/kg once every 19 days	Dectin-1	Dendritic Cells (DCs), Macrophages, T cells	Systemic tumor-antigen specific T cell response, increased infiltration of activated T cells into tumor, decreased immunosuppressive cells (Tregs, MDSCs).	↑ IL-12, ↑ IFN-γ, ↑ CD80/CD86/MHC-II on DCs, ↑ CD8^+^ T cell infiltration, ↓ Tregs, ↓ MDSCs, ↓ IL-10, ↓ TGF-β in tumor.	[95]
*Grifola frondosa* (Maitake Z-Fraction (MZF, Heteropolysaccharide) Injection of DCs treated with MZF (400 µg/mL)	Unknown (likely Dectin-1/TLR)	Dendritic Cells (DCs)	DC maturation, enhanced antigen presentation, induction of antigen-specific Th1 response.	↑ CD80, ↑ CD86, ↑ CD83, ↑ MHC-II on DCs; ↑ IL-12 and TNF-α production by DCs; ↑ antigen-specific IFN-γ production by CD4^+^ T cells (IL-12 dependent).	[92]
*Sanghuangporus vaninii*—Purified polysaccharides	Unknown (likely TLR4)	Dendritic Cells (DCs), CD4+ T cells	DC-mediated antigen presentation, Th1 differentiation.	↑ IFN-γ, ↑ TNF-α from Th1 cells, enhanced tumor sensitivity to CTLs.	[52]
*Trametes robiniophila* (Huaier)—Polysaccharides i.p., 60 mg/kg, once daily	TLR4	Macrophages	Macrophage activation.	↑ NO, ↑ TNF-α, ↑ IL-6 via TLR4-NF-κB/MAPK.	[96]

Abbreviations: DCs: Dendritic Cells; MDSCs: Myeloid-Derived Suppressor Cells; CTLs: Cytotoxic T Lymphocytes; i.p.: intraperitoneal injection. “↑”—increase; “↓”—decrease.

**Table 2:** Some *in vitro* and *in vivo* studies of mushroom-derived β-glucan-mediated antineoplastic activities in colorectal cancer models.

Substance/ Mushroom	Type of Research	Object	Effect	Reference
Hot water extract of *Inonotus obliquus*	*In vitro*	Colon cancer HT-29 cell line	The IC_50_ was approximately 1.0 mg/mL after a 48h treatment. Induction of the intrinsic pathway of apoptosis.	[111]
The neutral polysaccharide from *Agaricus bisporus*	*In vitro*	Colon cancer HT-29 cell line	Inhibition of proliferation and epithelial–mesenchymal transition, as well as induction of the intrinsic pathway of apoptosis.	[112]
Polysaccharide from *Cantharellus cibarius*	*In vitro*	Colon cancer cell line LS180 and colon epithelial cells CCD841	Selectivity against colon cancer cells; induction of cell cycle arrest and DNA fragmentation; inhibition of the NF-κB signaling pathway.	[113]
Selenium-enriched *Pleurotus ostreatus*	*In vitro*	Colon cancer HCT-116 cell line and normal mucosal epithelial cells NCM460	Selectivity against colon cancer cells; suppression of epithelial–mesenchymal transition; induction of apoptosis.	[114]
Polysaccharide-rich extracts from *Trametes versicolor* and *Grifola frondosa*	*In vitro*	LoVo and HT-29 colon cancer cells lines	Decreased proliferation, migration, and invasion capabilities; sensitization of colon cancer cells to 5-FU	[115]
Polysaccharides and proteoglycans from *Boletus edulis*	*In vitro*	Colon cancer cell line LS180 and colon epithelial cells	Selectivity against colon cancer cells; G0/G1 cell cycle arrest associated with the modulation of p16/cyclin D1/CDK4-6/pRb pathway	[116]
Polysaccharides from 53 wild-growing mushrooms	*In vitro*	Several Lactobacillus strains	The stronger stimulation of *Lactobacillus* growth than by commercially available prebiotics like inulin or fructooligosaccharides	[117]
Water-soluble polysaccharides from *Gloeostereum incarnatum*	*In vivo*	Apc^MinC^/Gpt mice (in situ colon cancer mouse model)	The eight-week administration of doses ranging from 30 to 90 mg/kg dramatically suppressed tumor growth, decreased IL-1*β*, IL-4, IL-6, IL-17, IL-22, and TNF-α, and mitigated the Wnt/β-catenin signaling pathway.	[118]
Polysaccharides from *Agaricus blazei*	*In vivo*	Syngeneic mouse model including colorectal adenocarcinoma MC38 cell line implanted into C57Bl/6 mice	Compared to 12-month-old tumor-bearing mice, ABMP has a stronger anti-tumor effect on 8-month-old mice. ABMP prevents weight change, lowers the amount of lipids in tumor tissues, and boosts the immune system.	[119]
Polysaccharides from *Lentinula edodes*	*In vitro* and *In vivo*	HT-29 cell line and HT-29 xenografts in nude mice	Induction of both intrinsic and extrinsic apoptotic pathways in vitro and in vivo; the inhibition of the NF-κB pathway; in vivo anticancer activity started from 0.2 mg/kg	[120]
Polysaccharides from *Phellinus linteus*	*In vitro* and *In vivo*	SW480 colon cancer and human umbilical vein endothelial cells (HUVEC); SW480 xenografts in athymic nude mice	A dose of 0.125–1 mg/mL inhibited proliferation, invasion, and motility, as well as down-regulating β-catenin and cyclin D levels. It also mitigated HUVEC proliferation and capillary tube formation, suppressed xenograft growth, and mitigated Wnt/β-catenin signaling in vivo.	[121]
*β*-glucans from *Armillaria mellea* and *Lentinula edodes*	*In vitro* and *In vivo*	Syngeneic mouse model including colon carcinoma CT26 cell line implanted into Balb/C mice	β-glucan reversed tumor-promoting M2-like macrophages into tumor-suppressing M1-like macrophages through the Akt/NF-κB and MAPK pathways. It also suppressed the viability of colon cancer cells in both in vitro and in vivo models.	[86]
Polysaccharides from *Sanghuangporus vaninii*	*In vivo*	B6/JGpt-Apcem1Cin (Min)/Gpt male (ApcMin/+) mice	Polysaccharides prevent dysbiosis of the gut microbiota and normalize their metabolic functions. They also improve antigen presentation in dendritic cells, activate CD4^+^ T cells, enhance Th1 differentiation, and increase IFN-γ and TNF-α. These cytokines target tumor cells and increase their susceptibility to cytotoxic T lymphocytes.	[52]
Polysaccharides from *Grifola frondosa*	*In vitro* and *In vivo*	Dendritic cells (DC); subcutaneous colon-26 tumor model	Polysaccharides induced the maturation of dendritic cells (DCs) and an antigen-specific Th1 response by enhancing IL-12 production by DCs. Activated DCs *in vivo* resulted in decreased tumor volume and prolonged survival.	[92]
*β*-glucans from *Lentinula edodes*	*In vitro* and *In vivo*	HT-29 and SW-480 cell lines; AOM/DSS model of colitis-associated colorectal carcinogenesis in mice	β-glucans displayed strong anti-inflammatory and anticancer activities in both in vitro and in vivo studies. They reconstructed the intestinal mucosal barrier, increased the content of short-chain fatty acids (SCFAs), regulated the metabolism of gut microbiota, and normalized the ratio of beneficial to detrimental microbiota.	[122]

**Table 3:** Some *in vitro* and *in vivo* studies on the antineoplastic activities of fungal immunomodulating proteins (FIPs).

Type of Neoplasia	Type of Research	Effect	Reference
**GMI (*Ganoderma microsporum* immunomodulating protein)**			
Lung cancer Syngeneic model: LLC1-bearing mice	*In vitro* and *in vivo*	GMI inhibits EMT and cell migration by disrupting cell adhesion and downregulating integrins, thus blocking focal adhesion kinase (FAK). GMI slows cell mobility by downregulating Slug through FAK inhibition. This increases epithelial-related markers, reduces metastatic lesions, and prolongs survival in LLC1-bearing mice.	[128]
Lung cancer (H1975 cells harboring EGFR L858R/T790M double mutation; Osimertinib-resistant H1975 cell line; xenografts)	*In vitro* and *in vivo*	GMI suppresses tumor growth and migration in drug-resistant lung cancer cells by targeting integrin proteins. Specifically, GMI inhibits the expression of integrins αV and β1. This, in turn, suppresses cancer stemness and metastasis in epidermal growth factor receptor (EGFR)-mutated, osimertinib-resistant lung cancer.	[129]
Pemetrexed-resistant lung cancer cells and xenografts	*In vitro* and *in vivo*	GMI induced autophagy and decreased the viability of pemetrexed-resistant lung cancer cells. Furthermore, GMI reduced stemness by downregulating CD133, CD44, NANOG, and OCT4. GMI also suppressed the growth of xenografts.	[109]
Oral carcinomas and xenografts	*In vitro* and *in vivo*	GMI induces cell death and inhibits key cancer stem cell (CSC) properties, including self-renewal, expression of the CSC markers ALDH1 and CD44, migration, and invasion, in oral carcinoma stem cells. Furthermore, GMI reduces tumor growth in mice. Mechanistically, GMI inhibits the IL-6/Stat3 signaling pathway.	[130]
Glioblastoma multiforme (GBM)	*In vitro*	GMI significantly suppressed the migration and invasion of glioblastoma cells. It also acted synergistically with the chemotherapy drug temozolomide to inhibit cell motility. This anti-cancer effect occurred through GMI inducing the degradation of Slug, a transcription factor linked to metastasis.	[131]
Lung cancer cell lines and xenografts	*In vitro* and *in vivo*	GMI inhibits the growth of lung cancer cells by binding directly to the epidermal growth factor receptor (EGFR), which blocks its dimerization and triggers clathrin-dependent endocytosis and degradation. This process suppresses tumor growth in mouse models and is effective against cancer cells with wild-type or mutant forms of EGFR.	[132]
Lung cancer cell lines; Syngeneic model: LLC1-bearing mice; and xenografts	*In vitro* and *in vivo*	GMI suppressed tumor growth in mouse models and inhibited KRAS activation, as well as the downstream MAPK and PI3K-AKT signaling pathways, in lung cancer cells with various KRAS mutations. Notably, GMI exhibited a strong synergistic effect with the KRASG12C inhibitor AMG 510, enhancing apoptosis and leading to more durable inhibition of tumor growth and KRAS activity.	[133]
**LZ-8**			
Lung cancer cell lines and xenografts	*In vitro* and *in vivo*	The recombinant protein LZ-8 inhibits the progression of lung cancer by binding to the epidermal growth factor receptor (EGFR), triggering its ubiquitination and subsequent degradation. This ultimately induces cell cycle arrest and apoptosis. This effect has been demonstrated in cancer cells with wild-type and mutated forms of EGFR, and it has been validated in a mouse model, in which LZ-8 suppresses tumor growth. LZ-8 mechanistically induces the formation of EGFR/Cbl complexes.	[134]
Lung cancer Syngeneic model: LLC1-bearing mice	*In vitro* and *in vivo*	LZ-8 suppressed tumor metastasis and increased survival in a mouse model of lung cancer by inhibiting the epithelial–mesenchymal transition (EMT) process. This was achieved by inactivating focal adhesion kinase (FAK), which enhanced the ubiquitination and proteasomal degradation of the key EMT transcription factor Slug by MDM2. The degradation of Slug increased the expression of E-cadherin, an adhesion protein that ultimately represses cancer cell mobility.	[135]
MBT-2 syngeneic model (Bladder cancer)	*In vivo*	The recombinant protein LZ-8 acted as a potent adjuvant, greatly enhancing the therapeutic effect of a HER-2/neu DNA vaccine against tumors in mice. It did so by stimulating dendritic cells via the TLR4 pathway. This boosted vaccine-induced Th1 and CTL immune responses, resulting in superior antitumor activity.	[136]
Lung cancer Syngeneic model: LLC1-bearing mice	*In vitro* and *in vivo*	Treatment with LZ-8 significantly altered the proteomic profile of lung tumors in mice, most notably by downregulating the expression of heat shock proteins (HSPs) 60, 70, and 90. LZ-8 and its homolog GMI were also shown to reduce HSP levels in vitro, subsequently inhibiting cell migration and inducing apoptosis.	[137]
Patients-derived hepatocellular carcinoma cell lines and xenografts	*In vitro* and *in vivo*	LZ-8 suppressed tumor progression and intrahepatic metastasis in patient-derived hepatocellular carcinoma (HCC) models regardless of c-Met signaling status. LZ-8 mechanistically inhibited cell migration by suppressing the c-Met axis in c-Met-positive tumors and the epidermal growth factor receptor (EGFR) axis in c-Met-negative tumors.	[138]

**Table 4:** *In vivo* studies of some low-molecular-weight compounds from mushrooms in cancer models.

Compound/Source	Type of Neoplasia/Type of Cancer	Effect	Reference
**Triterpenoids**			
Betulinic acid from *Inonotus obliquus*	4T1 breast cancer mouse model	Suppressed 4T1 tumor growth and blocked formation of pulmonary metastases without obvious side effects. Furthermore, histological and immunohistochemical analyses showed a decrease in MMP-9 positive cells, MMP-2 positive cells and Ki-67 positive cells and an increase in cleaved caspase-3 positive cells upon BA administration. Notably, BA reduced the number of myeloid-derived suppressor cells (MDSCs) in the lungs and tumors.	[178]
	KB cells in BALB/c mice	BA dose-dependently reduced implanted tumor volume and induced mitochondrial apoptosis, as shown by increased TUNEL^+^ cells, caspase-3/9 activity, Bax expression, and decreased Bcl-2. BA also elevated ROS and p53 levels in tumor tissues, and NAC or p53 knockdown diminished these effects, indicating that BA suppresses tumor growth through ROS-p53-mediated apoptotic signaling.	[179]
Betulin from *Inonotus obliquus*	CT26 lung metastasis model in BALB/c mice	Suppress the lung metastasis of CRC cells by inducing cell cycle arrest, apoptosis, and autophagy	[190]
Ganoderic acid T from *Ganoderma lucidum*	ES-2 orthotopic ovarian cancer model in a humanized mouse model	Significantly demonstrate cytotoxicity against various cancer cell lines and effectively remodeled the TME by reducing the α-SMA+ cell proportions, enhancing immune cell infiltration, and downregulating Gal-1 levels in the ES-2 orthotopic ovarian tumor model. Inhibition of Gal-1 expression via ubiquitination-induced protein degradation	[218]
	Lewis Lung Carcinoma (LLC) bearing C57B/6 mice	Demonstrate that GA-T suppresses tumor growth and LLC metastasis and down-regulates MMP-2 and MMP-9 mRNA expression	[317]
Ganoderic acid A from *Ganoderma lucidum*	EL4 syngeneic mouse model of metastatic lymphoma	Significantly prolonged survival of EL4 challenged mice and decreased tumor metastasis to the liver, an outcome accompanied by a marked down-regulation of STAT3 phosphorylation, reduction myeloid-derived suppressor cells (MDSCs), and enhancement of cytotoxic CD8+ T cells in the host	[318]
	Female BALB/c nude mice A549 cells-bearing	Enhanced the DDP-induced increase in E-cadherin and further reduced N-cadherin and vimentin levels in tumor tissues, indicating a stronger inhibition of EMT. Additionally, GA-A reversed the DDP-induced upregulation of Beclin and LC3II/LC3I, showing suppression of autophagy in A549/DDP-derived tumors.	[319]
Inotodiol and trametenolic acid-enriched fractions of *Inonotus obliquus*	4T1 breast cancer mouse model	Significantly reduced tumor volume; suppressed mTOR signaling; induced AMPK-dependent autophagy; did not compromise cytotoxicity of conventional drugs	[164]
Inotodiol from *Inonotus obliquus*	Diabetic rat model with induced breast cancer	Reduced PCNA positivity; increased apoptotic cells; decreased β-catenin, c-Myc, and Cyclin D1; improved blood glucose, cholesterol, triglycerides, HDL levels, and glucose tolerance	[163]
	P388 leukemia-bearing CDF1 mice	Intraperitoneal administration at 10 mg/kg significantly prolonged survival. %ILS: 20.8% at 10 mg/kg; 9.7% at 3 mg/kg. No apparent toxicity (no weight loss, no diarrhea).	[160]
**Diterpenoids**			
Erinacine A from *Hericium erinaceus*	Female athymic BALB/c-nu mice HCT-116 cells bearing	Significantly reduced tumor aggressiveness by decreasing cell proliferation and invasiveness, accompanied by increased ROS production and activation of the PI3K/mTOR/p70S6K and ROCK1/LIMK2/Cofilin signaling pathways.	[226]
	DLD-1 cells bearing female athymic BALB/c-nu mice	Suppressed tumor growth in the xenograft mouse model, as evidenced by a significant increase in TNFR, Fas, and FasL expression and a clear enhancement of apoptosis within tumor tissues.	[228]
Erinacine S from *Hericium erinaceus*	AGS cells bearing male athymic BALB/c-nu mice	Significantly reduced tumor burden while increasing FasL and TRAIL expression and lowering PCNA and cyclin D1 levels in xenograft tumors.	[229]
**Sesquiterpenoids**			
Grifolin from *Albatrellus confluens*	A549 cells bearing male BALB/c nude mice	Decreased CDK4, CDK6, and CyclinD1 expression and significantly decreased PIK3CA and p-AKT expression in lung cancer cells.	[235]
	5–8F-Z cells bearing BABL/c nude mice	Suppressed lung metastasis in the 5–8F-Z metastatic mouse model, reducing the metastatic incidence from 60% (6/10) in the control group to 18.2% (2/11) following 25 days of daily treatment at 32 mg/kg, without observable adverse effects.	[234]
**Phenolics and other compounds**			
Antroquinonol from *Antrodia camphorata*	Phase I clinical trial: patients with metastatic non-small-cell lung cancer (NSCLC) who had failed ≥2 prior treatments	Safe and well tolerated at 50–600 mg daily. No dose-limiting toxicities (DLTs) observed; maximum tolerated dose (MTD) not reached. Best tumor response: stable disease in 3 patients; no treatment-related mortality. Pharmacokinetics: rapid absorption (T_max_ 1–4 h), short half-life (1.3–4.33 h).	[254]
	C6 rat glioma xenograft in nude mice	Significantly reduced C6 glioma xenograft tumor volume without affecting body weight, and histological analysis showed extensive tumor cell death with no detectable toxicity in major organs, indicating potent and well-tolerated antitumor activity.	[253]
Cordycepin from *Cordyceps militaris*	Male nude BALB/c mice bearing CAL-27 cells	Markedly suppressed tumor growth, reducing tumor weight by up to 88.2% and increased apoptosis while lowering Ki67 and Bcl-2 levels	[320]
	BxPC-3 cells bearing female BALB/cA nu/nu mice	Significantly suppressed xenograft tumor growth and weight without affecting body weight, while reducing Ki67 staining and downregulating Ras and ERK phosphorylation, indicating an antitumor effect mediated through inhibition of the FGFR/Ras/ERK pathway	[321]
Hispolon from *Phellinus linteus*	DBTRG cells bearing NOD-SCID mice	Hispolon administered at 5 and 10 mg/kg every two days for 25 days markedly inhibited tumor growth, with the 10 mg/kg dose almost completely suppressing tumor expansion, and tumor tissues showing strong induction of cleaved caspase-3	[259]
	HL-60 cells bearing NSG mice	Hispolon (5 and 10 mg/kg) markedly suppressed tumor growth, reducing tumor weight by 29% and 34% and lowering Ki67 and procaspase-3 levels, indicating apoptosis-mediated antitumor activity in vivo.	[260]

## Data Availability

No new data were created or analyzed in this study. Data sharing is not applicable to this article.
